# Time-dose response and mechanistic specificity of berberine in renal fibrosis from a multi-model integration perspective: a systematic review and meta-analysis on animal models

**DOI:** 10.3389/fphar.2025.1600408

**Published:** 2025-06-11

**Authors:** Ruyi Nie, Ziting Yuan, Yizhe Wu, Minqi Pan, Jiandong Lu, Guoliang Xiong

**Affiliations:** ^1^ The Fourth Clinical Medical College of Guangzhou University of Chinese Medicine, Shenzhen, China; ^2^ Department of Nephrology, ShenZhen Traditional Chinese Medicine Hospital, Shenzhen, China

**Keywords:** berberine, renal fibrosis, mechanism of action, meta-analysis, systematic review, animal model

## Abstract

**Objective:**

This research intends to comprehensively evaluate the efficacy of berberine (BBR) and the specificity of its mechanisms of action in different animal models of renal fibrosis through a multi-model integration strategy.

**Methods:**

A comprehensive search of animal experimental studies was carried out across 10 different databases, including PubMed, Embase, Web of Science, Scopus, Cochrane Library, SCIELO, CNKI, Wanfang database, CBM and VIP Information Chinese Periodical Service Platform, spanning from their inception up to November 2024. The included studies’ methodological quality was assessed using the SYRCLE’s risk of bias tool for animal experiment, and statistical analyses were carried out with Stata 18.0.

**Results:**

In total, 26 animal studies (2010–2024) were included, encompassing diverse models of renal fibrosis. The Meta-analysis revealed that BBR significantly lowered serum creatinine, blood urea nitrogen, α-SMA, and TGF-β1 levels, alongside reductions in renal fibrosis area and oxidative stress markers. The time-dose response analysis indicated that BBR was most efficacious within the 100–400 mg/kg dose range over a 5–12-week intervention period. Still, the mechanism of action was model-dependent: in the UUO model, BBR predominantly modulated the AMPK/PPARα pathway and ferroptosis, while in the DN model, it primarily targeted glycolipid metabolism and epigenetic regulation.

**Conclusion:**

BBR significantly ameliorates renal fibrosis progression through a multi-targeted mechanism that is model-specific. Although preclinical evidence supports its therapeutic potential, the interpretation of the conclusions requires caution, considering the significant heterogeneity and methodological quality differences among the included experiments.

**Systematic Review Registration:**

https://www.crd.york.ac.uk/, identifier CRD42024619202

## 1 Introduction

Recognized as a significant worldwide health issue, chronic kidney disease (CKD) affects roughly 13.4% of the world’s population. Its elevated prevalence of disability and mortality from end-stage renal failure necessitates urgent development of effective intervention strategies (Lv and Zhang, 2019). The core pathological characteristic of CKD is renal fibrosis. Clinical evidence and renal biopsy data show a strong correlation between the severity of histopathological fibrosis and the rate of kidney function decline in end-stage renal disease patients (Sun et al., 2024), emphasizing the central role of renal fibrosis in CKD progression. This pathological process is marked by the abnormal extracellular matrix (ECM) buildup, aberrant activation of myofibroblasts, and disruption of the microvascular network, and induces structural alterations of the renal parenchyma. These alterations manifest as glomerular sclerosis, tubular atrophy, and interstitial fibrosis, ultimately leading to irreversible kidney function impairment (Yuan et al., 2019; Panizo et al., 2021; Djudjaj and Boor, 2019; Huang et al., 2023). Although single-cell sequencing and epigenetic studies have revealed critical molecular mechanisms driving renal fibrosis, such as dysregulation of the Notch/Wnt signaling pathway, cellular senescence-associated secretory phenotypes, and imbalance of the immune microenvironment, specific therapies to directly block the fibrotic progression are lacking in the healthcare practice (Yamashita and Kramann, 2024; Huang et al., 2023; Xu et al., 2020). Existing treatments, including renin-angiotensin system (RAS) inhibitors, mineralocorticoid receptor antagonists (MRA), and sodium-glucose cotransporter 2 (SGLT2) blockers, can slow down the advancement of CKD, but their antifibrotic effects are limited and subject to significant individual differences (Cooper et al., 2023; Folkerts et al., 2023; Dekkers and Gansevoort, 2020). Additionally, research indicates that the fibrotic microenvironment can hinder drug delivery and efficacy. As a result, the kidneys show reduced responsiveness to conventional treatments, which exacerbates treatment resistance (Nastase et al., 2018). These factors highlight the imperative need for novel therapeutic targets and strategies.

Natural compounds have shown unique advantages in organ fibrosis interventions due to their multi-target regulatory capacities. Berberine (BBR), an isoquinoline alkaloid, has been shown in preclinical studies to alleviate fibrosis in multiple organs, including the liver and lungs, by inhibiting inflammatory responses, regulating cellular autophagy, and metabolic reprogramming (Liu et al., 2024). Studies in the domain of renal fibrosis have proposed that it can alleviate renal pathological injury through multiple targets and mechanisms (Gao et al., 2024). However, the available evidence shows significant heterogeneity. The description of BBR’s mechanism in renal fibrosis across different animal models (e.g., ischemia-reperfusion, unilateral ureteral obstruction, and diabetic nephropathy) lacks cross-model integration analysis. Moreover, the time-dose response relationship remains poorly characterized. Notably, no comprehensive meta-analysis has yet synthesized preclinical evidence to systematically evaluate BBR’s efficacy against renal fibrosis. For this reason, This research employs a multi-model integration strategy to offer a scientifically grounded justification for the translational medicine application of BBR, and construct a theoretical framework for the precise therapeutic strategy of multi-targeted natural medicines in fibrotic diseases.

## 2 Materials and methods

This research was conducted in accordance with the PRISMA guidelines while following established methodological standards for systematic reviews. The complete study protocol is publicly accessible through the PROSPERO registry platform (https://www.crd.york.ac.uk/PROSPERO/view/CRD42024619202).

### 2.1 Search methodology

To identify pertinent animal studies, two independent authors searched 10 databases, including PubMed, Embase, Web of Science, Scopus, SCIELO, Cochrane Library, CNKI, Wanfang Database, CBM and VIP, covering records from database inception through November 2024, with language restrictions applied to English and Chinese publications. Detailed search strategies are provided in Supplementary Table S1.

### 2.2 Study selection criteria

Specified inclusion criteria established beforehand were: (1) Participants: animal models of renal fibrosis exhibiting pathological histological changes consistent with renal fibrosis; (2) Intervention: The treatment group received varying doses of BBR; (3) Control: The model group received either a placebo or no treatment; (4) Outcomes: The principal outcome indicators included serum creatinine (Scr), blood urea nitrogen (BUN), transforming growth factor-β1 (TGF-β1), and α-smooth muscle actin (α-SMA). Further outcome indicators included 24-h urinary protein, kidney weight index (KWI), renal fibrosis area, kidney injury molecule-1 (KIM-1), renal injury score, Collagen IV, Collagen I, fibronectin (FN), E-cadherin, superoxide dismutase (SOD), malondialdehyde (MDA), NOD-like receptor protein 3 (NLRP3), glutathione peroxidase (GSH-Px), and interleukin-1β (IL-1β).

The pre-specified exclusion criteria were: (1) studies focusing exclusively on cellular models or clinical research; (2) non-renal fibrosis models; (3) redundant or overlapping publications; (4) reviews; (5) conferences proceedings, dissertations, and thesis presentations; (6) case reports; (7) incomplete data; and (8) studies with unavailable full text.

### 2.3 Data extraction

Based on the predefined selection criteria, two independent assessors evaluated the study titles, summaries, and full manuscripts to identify those suitable for inclusion. When discrepancies occurred, we resolved them by discussing with another researcher. A standardized pretest form (Excel) was utilized to obtain relevant information from the selected studies for evidentiary integration. The extracted information included: first author, publication year, fundamental features of the experimental animals (including species, gender, sample size, and weight), intervention specifics (administration route, dose, duration), modeling methodology, histological staining method for renal pathology, outcome measures, and intergroup differences. Data were included exclusively corresponding to the maximum dose of BBR administered. For outcomes with multiple reported time points, we chose to analyze data from the longest observation period. When the data essential for us were presented merely in graphical format, we used Web Plot Digitizer (https://apps.automeris.io/wpd4/) to derive relevant data from the charts.

### 2.4 Assessment of bias risk

Two investigators separately evaluated the methodology’s quality in the incorporated studies by means of the SYRCLE tool for animal experiments (Hooijmans et al., 2014). This tool encompasses various types of biases, categorized as follows: 1) Selection Bias, which includes aspects such as the generation of sequences, baseline characteristics, and concealment of allocation; 2) Performance Bias, involving the random assignment of animals and ensuring that caregivers and investigators are blinded; 3) Detection Bias, which encompasses randomization for evaluating outcomes and the blinding of those assessing results; 4) Attrition Bias, stemming from incomplete outcome data; 5) Selective Reporting Bias; and 6) other biases, totaling ten distinct categories. If ambiguities arise, they were addressed by engaging in deliberation with an independent third-party investigator.

### 2.5 Statistical analysis

The evaluation was conducted utilizing STATA 18.0. Dichotomous variables were analyzed for intervention effects using the risk ratio (RR) and its 95% confidence interval (CI). For continuous variables, we measured the standardized mean difference (SMD) and their corresponding 95% CI. Statistical significance was established at P-value <0.05. Heterogeneity across study outcomes was quantitatively assessed using the χ^2^ test in conjunction with the I^2^ test. For study outcomes with low heterogeneity (I^2^ ≤ 50%), a fixed-effects model was used, whereas a random-effects model was utilized for those with substantial heterogeneity (I^2^ > 50%). To ensure the reliability of the outcomes, we carried out sensitivity analyses on indicators with significant heterogeneity (I^2^ > 50%) by excluding studies individually to assess their impact on the combined effect.

### 2.6 Subgroup analysis

The primary outcome indicators were analyzed through subgroup evaluations to recognize potential factors contributing to heterogeneity among the studies included. The evaluations were categorized by modeling techniques, publication periods (pre-2017 and post-2017), animal species (mice versus rats), drug dosages (≤200 mg/kg and >200 mg/kg), and treatment duration (>8 weeks and ≤8 weeks). A P-value <0.05 established statistical significance.

### 2.7 Publication bias

STATA 18.0 software was used to draw funnel plots. To assess publication bias, we examined the symmetry of the funnel plots as a primary indicator. Additionally, the Egger test can identify potential publication bias, with significance at P < 0.05.

## 3 Result

### 3.1 Literature search results

**FIGURE 1:**
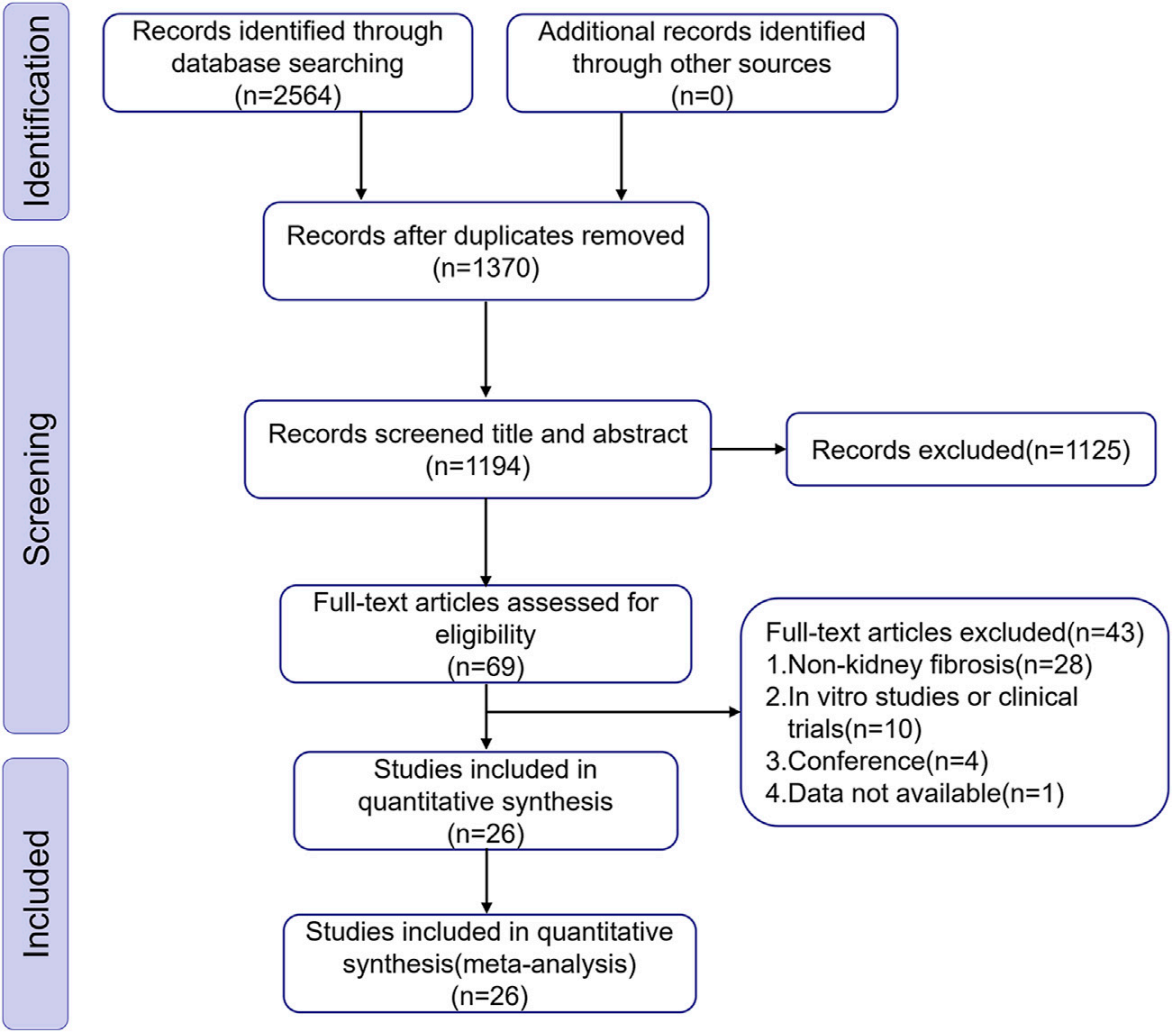
Diagram illustrating the study inclusion procedure.

We discovered 2,564 articles of potential relevance from ten databases. After removing 1,370 duplicate records, 1,194 articles remained for additional screening. Subsequently, 1,168 articles were excluded through title/abstract evaluation and full-text review according to predefined exclusion criteria. Ultimately, We incorporated 26 eligible studies covering the period from 2010 to 2024, reflecting a growing research interest in BBR’s therapeutic efficacy against renal fibrosis in recent years. Figure 1 illustrates the detailed process of selection.

### 3.2 Characteristics of the included studies

This study encompassed 26 animal experiments (2010–2024) involving 472 renal fibrosis model animals, split evenly between a treatment group of 236 and a control group of 236. The experiments were conducted using mice and rats as the animal models, such as Sprague Dawley rats in 11 studies (42.3%), C57BL/6 mice in 7 studies (26.92%), Wistar rats in 5 studies (19.23%), and one study each using KKAy mice (3.85%), db/db mice (3.85%) and Albino rats (3.85%). Sex distribution was dominated by male animals (88.5%); 2 studies (7.7%) used female animals, and only 1 study (3.8%) did not specify sex. Most studies reported the initial weight of the animals, although five studies did not specify a weight range. Regarding model construction, the diabetic nephropathy (DN) model was the predominant induction type, accounting for 17 studies (69.2%). Among these, 14 models were induced using streptozotocin (STZ), either alone or in combination with a high-fat/high-sugar diet, while one model used alloxan. The remaining models included unilateral ureteral obstruction (UUO, 4 studies), unilateral renal artery stenosis (1 study), renal ischemia-reperfusion (I/R, 1 study), and drug-induced models (e.g., doxorubicin, cisplatin, adenine, totaling 3 studies). The dosage of BBR administered ranged from 25 to 400 mg/kg, with treatment durations ranging from 2 to 20 weeks. Regarding outcome measures, Scr levels were reported in 25 studies; BUN levels in 22 studies; TGF-β1 levels in 12 studies; α-SMA levels in 8 studies; 24-h urinary protein levels in 10 studies; KWI in 14 studies; renal fibrosis area in 8 studies; Collagen I in 5 studies; Collagen IV levels in 6 studies; FN levels in 8 studies; E-cadherin levels in 4 studies; KIM-1 levels in 3 studies; and renal injury scores in 5 studies. Several studies also measured inflammatory markers, like NLRP3 and IL-1β, along with oxidative stress parameters, including GSH-Px, MDA, and SOD. Renal histopathological assessment was dominated by hematoxylin and eosin (H&E), Masson trichrome, and Periodic Acid-Schiff (PAS) staining. Table 1 details the included studies’ characteristics.

**TABLE 1:** Characteristics of the included studies.

Study (year)	Species (sex,n = treatment/model group, weight)	Modeling method	Intervention (administration, dosage, duration)	Renal histopathology	Outcomes	Intergroup differences
Liu et al. (2010)	C57BL/6 mice (male,8/8,23 ± 2 g)	Alloxan-induced DN models	By Intragastric 300 mg/kg; 12 weeks	—	1.Scr; 2.BUN; 3.TGF-β1 4.KWI; 5.FN	1.P < 0.05; 2.P < 0.05 3.P < 0.05; 4.P < 0.05 5.P < 0.05
Huang et al. (2012)	Sprague Dawley rats (male,10/10,210 ± 20 g)	STZ-induced DN models	By Intragastric 200 mg/kg; 12 weeks	—	1.Scr; 2.BUN; 3.KWI 4.FN	1.P < 0.05; 2.P < 0.05 3.P < 0.05; 4.P < 0.05
Liu et al. (2012)	Sprague Dawley rats (male,14/14,200–250 g)	STZ-induced DN models	By Intragastric 50/100/200 mg/kg; 5 weeks	H&E staining; Masson’s trichrome staining	1.Scr; 2.BUN; 3.TGF-β1 4.24 h urinary protein	1.P < 0.05; 2.P < 0.01 3.P < 0.01; 4.P < 0.05
Xie et al. (2013)	Sprague Dawley rats (male,8/8,200 ± 10 g)	STZ-induced DN models	By Intragastric 200 mg/kg; 12 weeks	PAS staining	1.Scr; 2.BUN; 3.TGF-β1 4.24 h urinary protein 5.KWI; 6.CollagenⅣ 7.FN; 8.MDA; 9.SOD	1.P < 0.05; 2.P < 0.05 3.P < 0.05; 4.P < 0.05 5.P < 0.05; 6.P < 0.05 7.P < 0.05; 8.P < 0.05 9.P < 0.05
Wang et al. (2014)	Sprague Dawley rats (male,8/8,180–220 g)	UUO models	By Intragastric 200 mg/kg; 2 weeks	H&E staining; Masson’s trichrome staining; Sirius red staining	1.Scr; 2.BUN; 3.TGF-β1 4.α-SMA; 5.KWI 6.Fibrotic area 7.Renal injury score 8.MDA; 9.SOD; 10.GSH-Px	1.P < 0.05; 2.P < 0.05 3.P < 0.05; 4.P < 0.05 5.P < 0.05; 6.P < 0.05 7.P < 0.05; 8.P < 0.05 9.P < 0.05; 10.P > 0.05
Miao and Zhang (2014)	Wistar rats (male,10/10,220–240 g)	Unilateral renal artery stenosis models	By Intragastric 100 mg/kg; 6 weeks	H&E staining; Masson’s trichrome staining	1.Scr; 2.TGF-β1	1.P < 0.05; 2.P < 0.05
Ni et al. (2015)	Sprague Dawley rats (male,10/10,180 ± 20 g)	STZ-induced DN models	By Intragastric 50/100/200 mg/kg; 8 weeks	—	1.Scr; 2.BUN; 3.TGF-β1 4.KWI; 5.Collagen-Ⅳ; 6.FN	1.P < 0.01; 2.P < 0.01 3.P < 0.01; 4.P < 0.01 5.P < 0.01; 6.P < 0.01
Sun et al. (2015)	Wistar rats (male,9/9,200–250 g)	DN models induced by STZ and high-fat diet	By Intragastric 25 mg/kg; 20 weeks	PAS staining; Masson’s trichrome staining	1.Scr; 2.BUN 3.CollagenⅠ 4.CollagenⅣ; 5.FN 6.Renal injury score	1.P > 0.05; 2.P > 0.05 3.P < 0.01; 4.P < 0.05 5.P < 0.05; 6.P < 0.05
Zhang et al. (2016)	C57BL/6J mice (—,8/8,20–25 g)	STZ-induced DN models	By oral administration 200 mg/kg; 12 weeks	—	1.Scr; 2.BUN; 3.α-SMA 4.24 h urinary protein 5.KWI; 6.CollagenⅠ	1.P < 0.01; 2.P < 0.01 3.P < 0.01; 4.P < 0.01 5.P < 0.05; 6.P < 0.01
Ma et al. (2016)	Sprague Dawley rats (male,10/10,160–180 g)	DN models induced by STZ and high-fat diet	By Intragastric 150 mg/kg; 8 weeks	H&E staining; Masson’s trichrome staining	1.Scr; 2.BUN; 3.α-SMA 4.24 h urinary protein 5.KWI; 6.E-cad 7.Fibrotic area	1.P > 0.05; 2.P > 0.05 3.P < 0.05; 4.P < 0.05 5.P < 0.05; 6.P < 0.05 7.P < 0.05
Qiu et al. (2017)	Sprague Dawley rats (male,12/12,200 ± 20 g)	DN models induced by STZ and high-fat and high-sugar diet	By Intragastric 50/100/200 mg/kg; 8 weeks	H&E staining PAS staining	1.Scr; 2.BUN; 3.TGF-β1 4.KWI	1.P < 0.001; 2.P < 0.05 3.P < 0.01; 4.P < 0.05
Yang et al. (2017)	KKAy mice (female,10/10,25–28 g)	DN models	By Intragastric 150 mg/kg; 16 weeks	H&E staining Mallory staining	1.Scr; 2.BUN; 3.α-SMA 4.24 h urinary protein 5.KWI; 6.E-Cad	1.P < 0.01; 2.P < 0.01 3.P < 0.01; 4.P < 0.01 5.P < 0.01; 6.P < 0.01
Li and Zhang (2017)	Wistar rats (male,10/10,150 ± 10 g)	STZ-induced DN models	By oral administration 400 mg/kg; 12 weeks	PAS staining; Masson’s trichrome staining	1.Scr; 2.BUN; 3.TGF-β1 4.α-SMA 5.24 h urinary protein	1.P < 0.05; 2.P < 0.05 3.P < 0.05; 4.P < 0.05 5.P < 0.05
Sun et al. (2020)	C57BL/6 mice (male,6/6,21–25 g)	I/R models	By Intragastric 100 mg/kg; 2 weeks	H&E staining; Masson’s trichrome staining	1.Scr; 2.BUN 3.Renal injury score 4.IL-1β; 5.MDA 6.SOD; 7.GSH-Px	1.P < 0.05; 2.P < 0.05 3.P < 0.05; 4.P < 0.05 5.P < 0.05; 6.P < 0.05 7.P < 0.05
Yu et al. (2020)	Sprague Dawley rats (male,10/10,200 ± 10 g)	adenine-induced CRF models	By Intragastric 150/300 mg/kg; 4 weeks	H&E staining	1.Scr; 2.BUN; 3.TGF-β1 4.α-SMA; 5.Collagen I 6.MDA; 7.SOD; 8.GSH-Px	1.P < 0.01; 2.P < 0.05 3.P < 0.05; 4.P < 0.05 5.P < 0.05; 6.P < 0.05 7.P < 0.05; 8.P < 0.05
Xiao et al. (2021)	db/db mice (male,10/10,—)	DN models	By Intragastric 100 mg/kg; 8 weeks	H&E staining PAS staining Masson’s trichrome staining	1.Scr; 2.BUN; 3.TGF-β1 4.24 h urinary protein 5.KWI	1.P < 0.01; 2.P < 0.01 3.P < 0.05; 4.P < 0.01 5.P > 0.05
Ibrahim Fouad et al. (2021)	Wistar rats (male,6/6,130 ± 20 g)	Doxorubicin induced renal fibrosis model	By oral administration 50 mg/kg; 2 weeks	H&E staining; Masson’s trichrome staining	1.Scr; 2.BUN; 3.TGF-β1 4.KWI; 5.Fibrotic area 6.KIM-1; 7.MDA	1.P ≤ 0.05; 2.P ≤ 0.05 3.P ≤ 0.05; 4.P ≤ 0.05 5.P ≤ 0.05; 6.P ≤ 0.05 7.P ≤ 0.05
Ma et al. (2022)	Sprague Dawley rats (male,10/10,—)	DN models induced by STZ and high-fat diet	By oral administration 150 mg/kg; 12 weeks	H&E staining; Masson’s trichrome staining	1.Scr; 2.BUN; 3.a-SMA 4.24 h urinary protein 5.KWI; 6.CollagenⅠ 7.CollagenⅣ; 8.FN 9.E-cad 10.Fibrotic area 11.KIM-1 12.Renal injury score 13.IL-1β; 14.NLRP3	1.P > 0.05; 2.P > 0.05 3.P < 0.05; 4.P < 0.05 5.P < 0.05; 6.P < 0.05 7.P < 0.05; 8.P < 0.05 9.P < 0.05; 10.P < 0.05 11.P < 0.05; 12.P < 0.05 13.P < 0.05; 14.P < 0.05
Ahmedy et al. (2022)	Wistar rats (female,10/10,170–200 g)	Cisplatin induced renal fibrosis model	By oral administration 100/200 mg/kg; 2 weeks	H&E staining Sirius red staining	1.Scr; 2.BUN 3.Fibrotic area 4.KIM-1	1.P < 0.05; 2.P < 0.05 3.P < 0.05; 4.P < 0.05
Tan et al. (2023)	C57BL/6 mice (male,6/6,—)	UUO models	By Intragastric 50 mg/kg; 2 weeks	PAS staining Masson’s trichrome staining	1.Scr; 2.BUN; 3.α-SMA 4.CollagenⅠ 5.CollagenⅣ; 6.FN 7.E-cad; 8.Fibrotic area 9.Renal injury score 10.IL-1β; 11.NLRP3	1.P < 0.05; 2.P < 0.05 3.P < 0.05; 4.P < 0.05 5.P < 0.05; 6.P < 0.05 7.P < 0.05; 8.P < 0.05 9.P < 0.05; 10.P < 0.05 11.P < 0.05
Al-Jebouri and Al-Murshidi (2023)	Albino rats (male,6/6,—)	STZ-induced DN models	By oral administration 200 mg/kg; 45 days	H&E staining	1.Scr; 2.BUN 3.24 h urinary protein	1.P < 0.05; 2.P < 0.05 3.P < 0.05
Wang et al. (2023)	Sprague Dawley rats (male,10/10,180 ± 20 g)	DN models induced by STZ and high-fat and high-sugar diet	— 50/100/200 mg/kg; 20 weeks	H&E staining	1.Scr	1.P < 0.05
Liu et al. (2023)	C57BL/6J mice (male,7/7,20–25 g)	UUO models	By Intragastric 200 mg/kg; 10 days	H&E staining; Masson’s trichrome staining	1.Fibrotic area	1.P < 0.05
Cai et al. (2024)	C57BL/6J mice (male,10/10,---)	STZ-induced DN models	By Intragastric 100/200 mg/kg; 8 weeks	H&E staining; Masson’s trichrome staining; PAS staining	1.Scr; 2.KWI 3.Fibrotic area 4.MDA; 5.GSH-Px	1.P < 0.001; 2.P < 0.01 3.P < 0.001; 4.P < 0.001 5.P < 0.01
Ma et al. (2024)	Sprague Dawley rats (male,10/10,160–180 g)	DN models induced by STZ and high-fat diet	By Intragastric 150 mg/kg; 12 weeks	H&E staining; Masson’s trichrome staining	1.Scr; 2.BUN 3.24 h urinary protein 4.KWI; 5.CollagenⅣ 6.FN; 7.IL-1β; 8.NLRP3	1.P > 0.05; 2.P > 0.05 3.P < 0.05; 4.P < 0.05 5.P < 0.05; 6.P < 0.05 7.P < 0.05; 8.P < 0.05
Li et al. (2024)	C57BL/6 mice (male,8/8,20–22 g)	UUO models	By Intragastric 50/100/200 mg/kg; 2 weeks	H&E staining; Masson’s trichrome staining TUNEL staining	1.Scr; 2.BUN; 3.TGF-β1 4.IL-1β	1.P < 0.01; 2.P < 0.01 3.P < 0.01; 4.P < 0.01

Scr: serum creatinine; BUN: blood urea nitrogen; TGF-β1: transforming growth factor-β1; α-SMA: α-smooth muscle actin; KWI: kidney weight index; KIM-1: kidney injury molecule-1; FN: fibronectin; E-cad: E-cadherin; SOD: superoxide dismutase; MDA: malondialdehyde; NLRP3: NOD-like receptor protein 3; GSH-Px: glutathione peroxidase; IL-1β:interleukin-1β; UUO: unilateral ureteric obstruction; DN:diabetic nephropathy; STZ:streptozotocin; I/R:renal ischemia-reperfusion; CRF: chronic renal failure; H&E: hematoxylin and eosin; PAS:Periodic Acid-Schiff.

### 3.3 Quality assessment

We evaluated the methodological quality of every included study based on the specified assessment criteria. Among the 26 incorporated studies, four studies (Ma et al., 2016; Sun et al., 2020; Ma et al., 2022; Ma et al., 2024) reported specific randomization; eighteen studies (Liu et al., 2010; Huang et al., 2012; Liu et al., 2012; Xie et al., 2013; Wang et al., 2014; Miao and Zhang, 2014; Sun et al., 2015; Zhang et al., 2016; Qiu et al., 2017; Yang et al., 2017; Li and Zhang, 2017; Yu et al., 2020; Xiao et al., 2021; Ibrahim Fouad and Ahmed, 2021; Tan et al., 2023; Al-Jebouri and Al-Murshidi, 2023; Liu et al., 2023; Li et al., 2024) mentioned random allocation without specifying the randomization method, while the remaining four studies (Ni et al., 2015; Ahmedy et al., 2022; Wang et al., 2023; Cai et al., 2024) lacked information on whether they were randomly grouped. Only one study (Tan et al., 2023) reported allocation concealment, while the rest did not. Twelve studies (Liu et al., 2012; Xie et al., 2013; Ni et al., 2015; Sun et al., 2015; Ma et al., 2016; Qiu et al., 2017; Li and Zhang, 2017; Xiao et al., 2021; Ibrahim Fouad and Ahmed, 2021; Ma et al., 2022; Ahmedy et al., 2022; Ma et al., 2024) described identical animal housing conditions; fourteen studies (Liu et al., 2010; Huang et al., 2012; Wang et al., 2014; Miao and Zhang, 2014; Zhang et al., 2016; Yang et al., 2017; Sun et al., 2020; Yu et al., 2020; Tan et al., 2023; Al-Jebouri and Al-Murshidi, 2023; Wang et al., 2023; Liu et al., 2023; Cai et al., 2024; Li et al., 2024) did not provide specifics. Six studies (Liu et al., 2012; Qiu et al., 2017; Yang et al., 2017; Wang et al., 2023; Cai et al., 2024; Li et al., 2024) failed to report complete data, while the other twenty studies reported complete data. None of the studies mentioned intervention blinding, randomization for outcome assessment, or outcome blinding. Each study described how they balanced intergroup baseline characteristics. There were a lack of selective reporting bias and other bias in all studies. Methodological quality evaluation can be found in Supplementary Table S2.

### 3.4 Effectiveness

#### 3.4.1 Principal outcome indicators

##### 3.4.1.1 Scr

**FIGURE 2:**
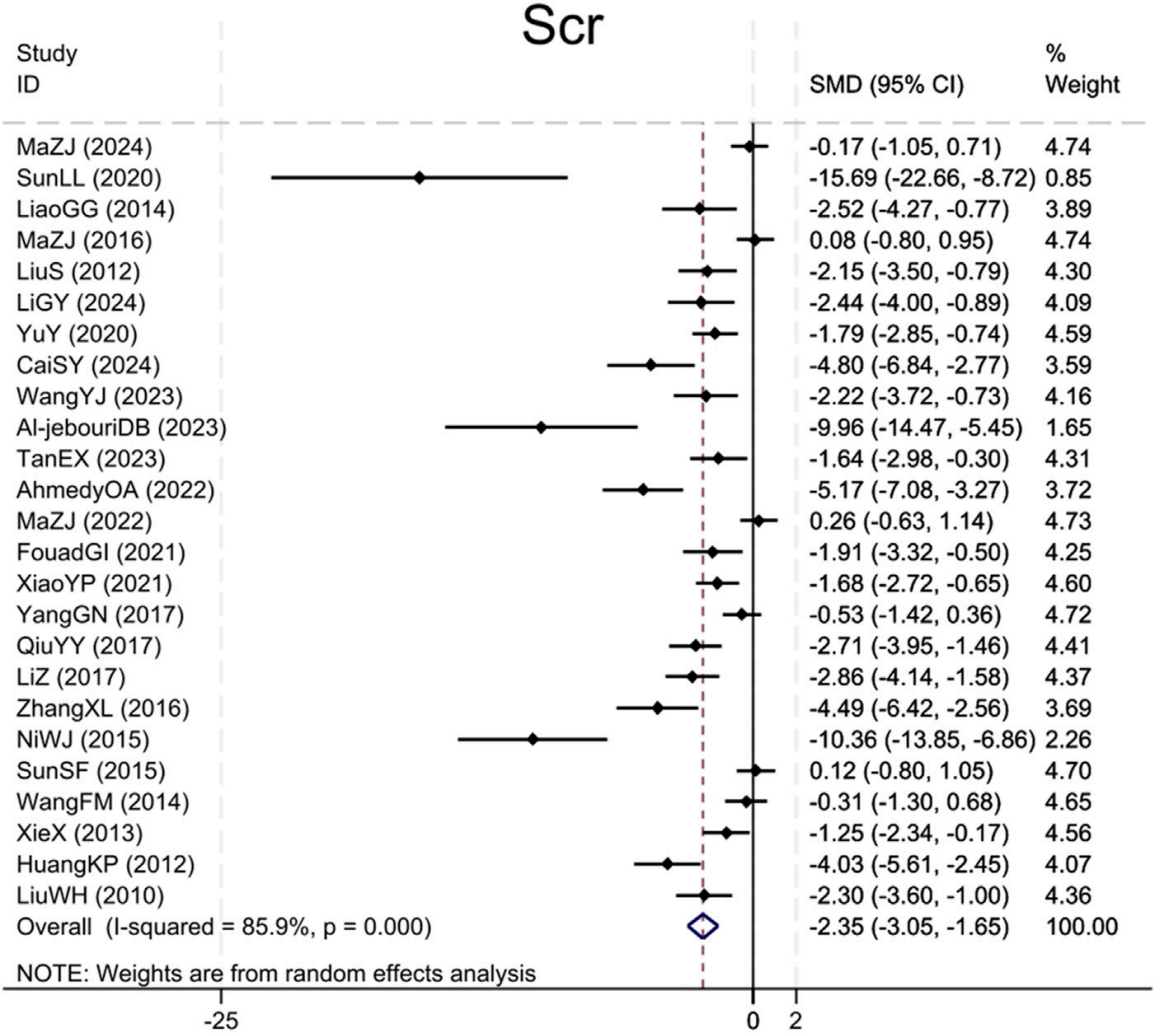
Forest plot for Scr.

Twenty-five studies (Liu et al., 2010; Huang et al., 2012; Liu et al., 2012; Xie et al., 2013; Wang et al., 2014; Miao and Zhang, 2014; Ni et al., 2015; Sun et al., 2015; Ma et al., 2016; Zhang et al., 2016; Qiu et al., 2017; Yang et al., 2017; Li and Zhang, 2017; Yu et al., 2020; Sun et al., 2020; Xiao et al., 2021; Ibrahim Fouad and Ahmed, 2021; Ahmedy et al., 2022; Ma et al., 2022; Tan et al., 2023; Al-Jebouri and Al-Murshidi, 2023; Wang et al., 2023; Cai et al., 2024; Ma et al., 2024; Li et al., 2024) reported Scr levels, demonstrating that the BBR group caused a significant Scr levels reduction compared to the model group (Sample size: 414; SMD = −2.35 (95% CI: −3.05 to −1.65), P < 0.001; χ^2^ = 170.62, I^2^ = 85.9%, Figure 2).

##### 3.4.1.2 BUN

**FIGURE 3:**
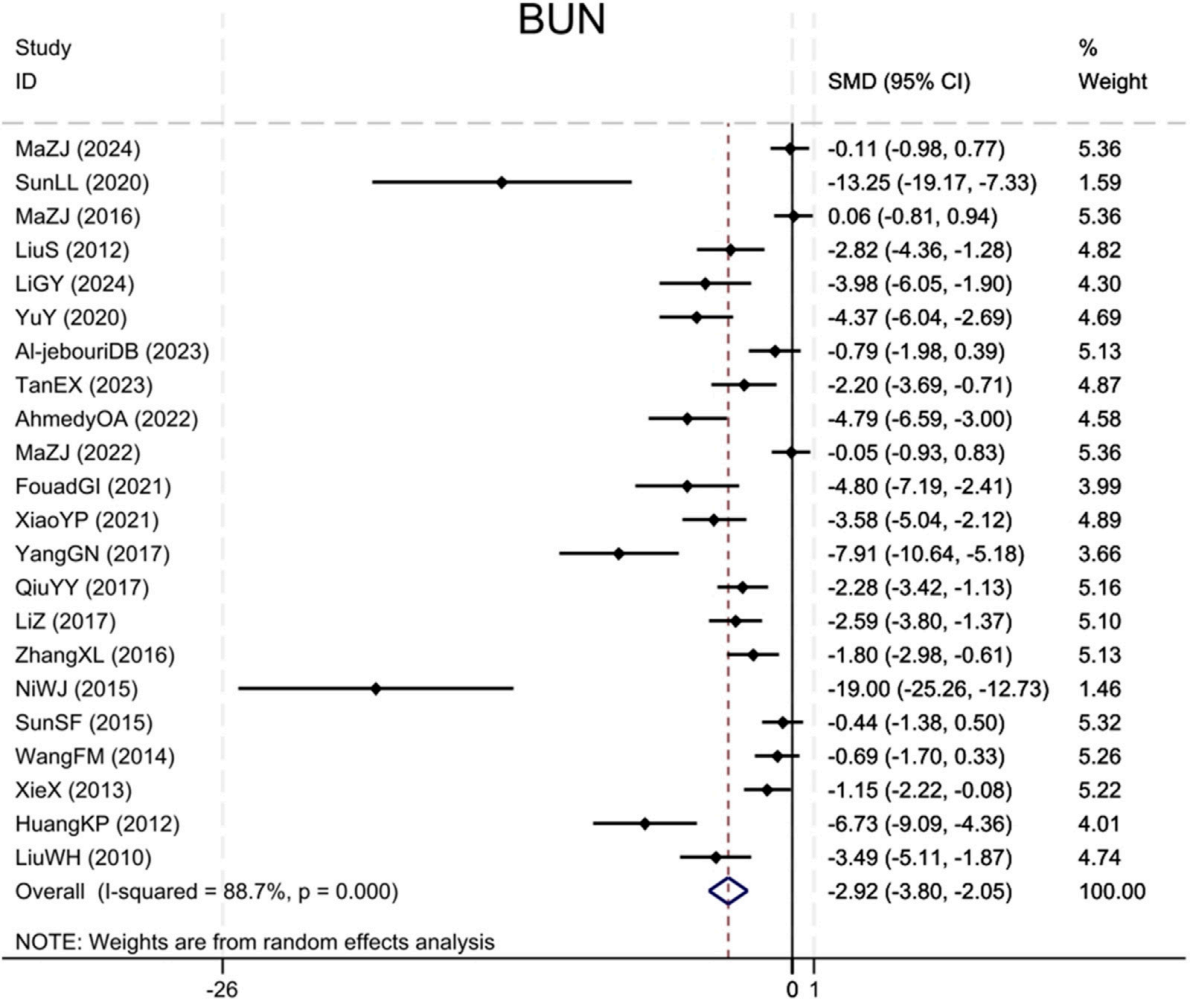
Forest plot for BUN.

Twenty-two studies (Liu et al., 2010; Huang et al., 2012; Liu et al., 2012; Xie et al., 2013; Wang et al., 2014; Ni et al., 2015; Sun et al., 2015; Ma et al., 2016; Zhang et al., 2016; Qiu et al., 2017; Yang et al., 2017; Li and Zhang, 2017; Yu et al., 2020; Sun et al., 2020; Xiao et al., 2021; Ibrahim Fouad and Ahmed, 2021; Ahmedy et al., 2022; Ma et al., 2022; Tan et al., 2023; Al-Jebouri and Al-Murshidi, 2023; Ma et al., 2024; Li et al., 2024) reported BUN levels, showing that the BBR group significantly decreased BUN as compared to the control group (Sample size: 376; SMD = −2.92 (95% CI: −3.80 to −2.05), P < 0.001; χ^2^ = 185.44, I^2^ = 88.7%, Figure 3).

##### 3.4.1.3 TGF-β1

**FIGURE 4:**
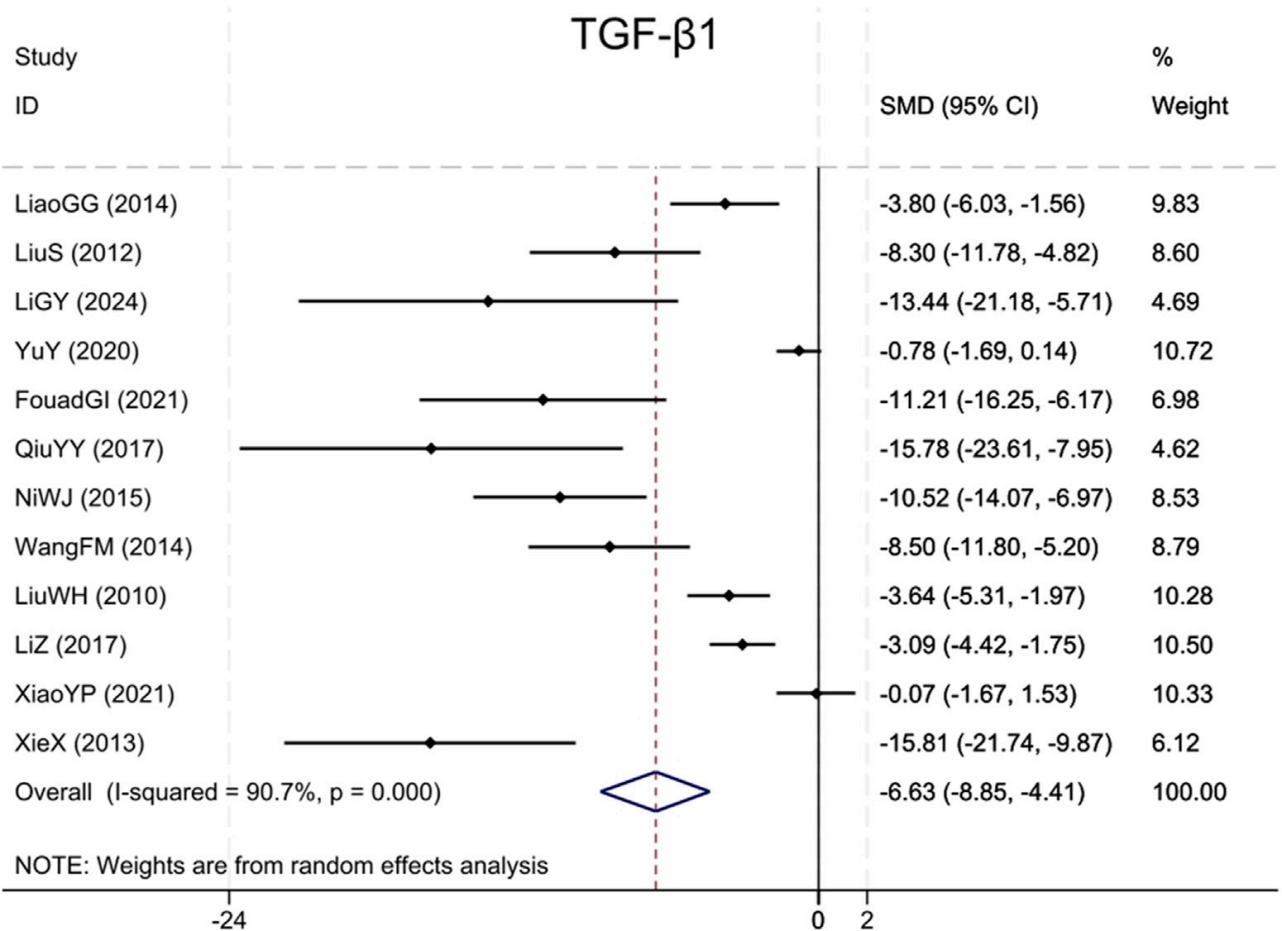
Forest plot for TGF-β1.

Analysis of twelve studies (Liu et al., 2010; Liu et al., 2012; Xie et al., 2013; Wang et al., 2014; Miao and Zhang, 2014; Ni et al., 2015; Qiu et al., 2017; Li and Zhang, 2017; Yu et al., 2020; Xiao et al., 2021; Ibrahim Fouad and Ahmed, 2021; Li et al., 2024) indicated that the BBR group exhibited markedly reduced TGF-β1 levels (Sample size: 168; SMD = −6.63 (95% CI: −8.85 to −4.41), P < 0.001; χ^2^ = 118.20, I^2^ = 90.7%, Figure 4).

##### 3.4.1.4 α-SMA

**FIGURE 5:**
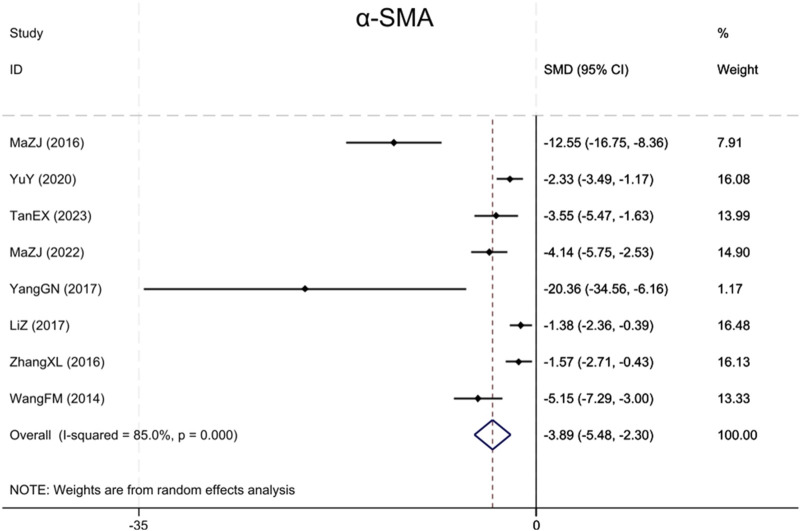
Forest plot for α-SMA.

Eight studies (Wang et al., 2014; Ma et al., 2016; Zhang et al., 2016; Yang et al., 2017; Li and Zhang, 2017; Yu et al., 2020; Ma et al., 2022; Tan et al., 2023) reported α-SMA, with results showing that BBR significantly reduced α-SMA levels more in renal fibrosis animals than in the model group (Sample size: 130; SMD = −3.89 (95% CI: −5.48 to −2.30), P < 0.001; χ^2^ = 46.77, I^2^ = 85.0%, Figure 5).

#### 3.4.2 Histopathological examination of the kidney

Histopathological examination was conducted in 22 studies. Among these, H&E staining was applied across 18 studies (Liu et al., 2012; Wang et al., 2014; Miao and Zhang, 2014; Ma et al., 2016; Qiu et al., 2017; Yang et al., 2017; Yu et al., 2020; Sun et al., 2020; Xiao et al., 2021; Ibrahim Fouad and Ahmed, 2021; Ahmedy et al., 2022; Ma et al., 2022; Al-Jebouri and Al-Murshidi, 2023; Liu et al., 2023; Wang et al., 2023; Cai et al., 2024; Ma et al., 2024; Li et al., 2024); Masson trichrome method was applied across 15 studies (Liu et al., 2012; Wang et al., 2014; Miao and Zhang, 2014; Sun et al., 2015; Ma et al., 2016; Li and Zhang, 2017; Sun et al., 2020; Xiao et al., 2021; Ibrahim Fouad and Ahmed, 2021; Ma et al., 2022; Tan et al., 2023; Liu et al., 2023; Cai et al., 2024; Ma et al., 2024; Li et al., 2024); PAS staining was used in 7 studies (Xie et al., 2013; Sun et al., 2015; Qiu et al., 2017; Li and Zhang, 2017; Xiao et al., 2021; Tan et al., 2023; Cai et al., 2024); Sirius red staining in two studies (Ahmedy et al., 2022; Wang et al., 2014); Mallory staining in one study (Yang et al., 2017); and TUNEL staining in one study (Li et al., 2024). Upon treatment with BBR, H&E staining demonstrated a notable reduction in renal tubular epithelial cell vacuolar degeneration, atrophy, and inflammatory cell infiltration, as well as an improvement in glomerular basement membrane thickening and mesangial hyperplasia, relative to the model groups. Masson and Sirius red staining together demonstrated that both renal fibrosis and collagen deposition showed a marked decrease, including the decrease in collagen fiber area and fibrous tissue content. Mallory staining further confirmed that BBR could alleviate glomerulosclerosis and mesangial fibrosis. PAS staining indicated that BBR reduced the extension of the mesangial matrix and glycogen deposition, and repaired the basement membrane structure. Additionally, TUNEL staining showed a reduction in renal tissue apoptosis after BBR treatment, suggesting its anti-apoptotic effect. Those findings suggest that BBR demonstrates a protective role in various kidney injury models through multiple mechanisms, encompassing anti-inflammatory, anti-fibrotic, anti-apoptotic, and structural repair. These results show its potential as a multi-target therapeutic agent.

#### 3.4.3 24 h urine protein

**FIGURE 6:**
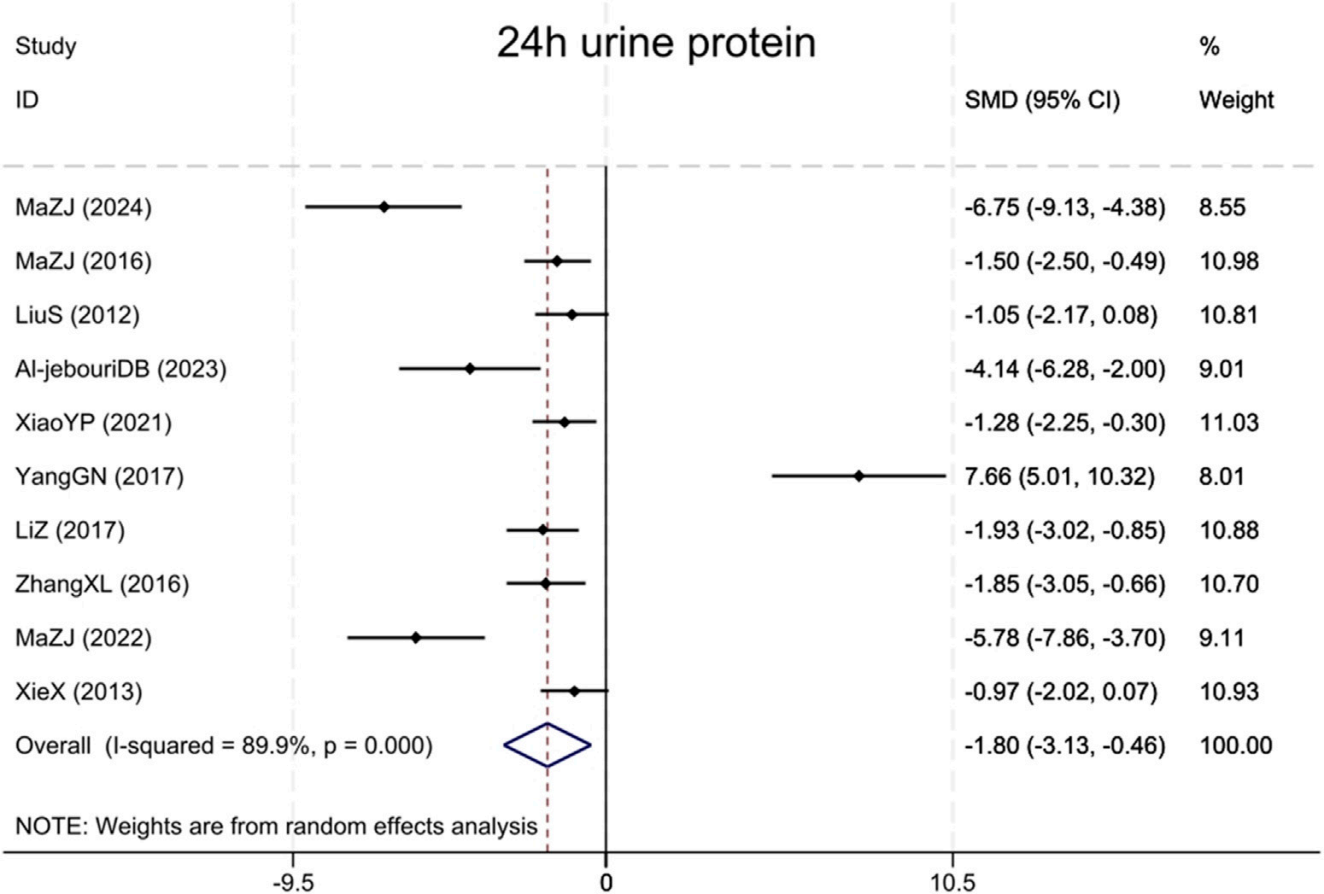
Forest plot for 24 h urine protein.

Assessment of proteinuria in the combined analysis consisted primarily of 24 h urine microalbumin and 24 h urine albumin, and these results are collectively referred to as 24 h urine protein. A total of 10 studies (Liu et al., 2012; Xie et al., 2013; Ma et al., 2016; Zhang et al., 2016; Yang et al., 2017; Li and Zhang, 2017; Xiao et al., 2021; Ma et al., 2022; Al-Jebouri and Al-Murshidi, 2023; Ma et al., 2024) assessed 24 h urinary protein, revealing that the BBR group had markedly reduced urinary protein excretion compared to the model group (Sample size: 178; SMD = −1.80 (95% CI: −3.13 to −0.46), P < 0.001; χ^2^ = 89.18, I^2^ = 89.9%, Figure 6).

#### 3.4.4 KWI

**FIGURE 7:**
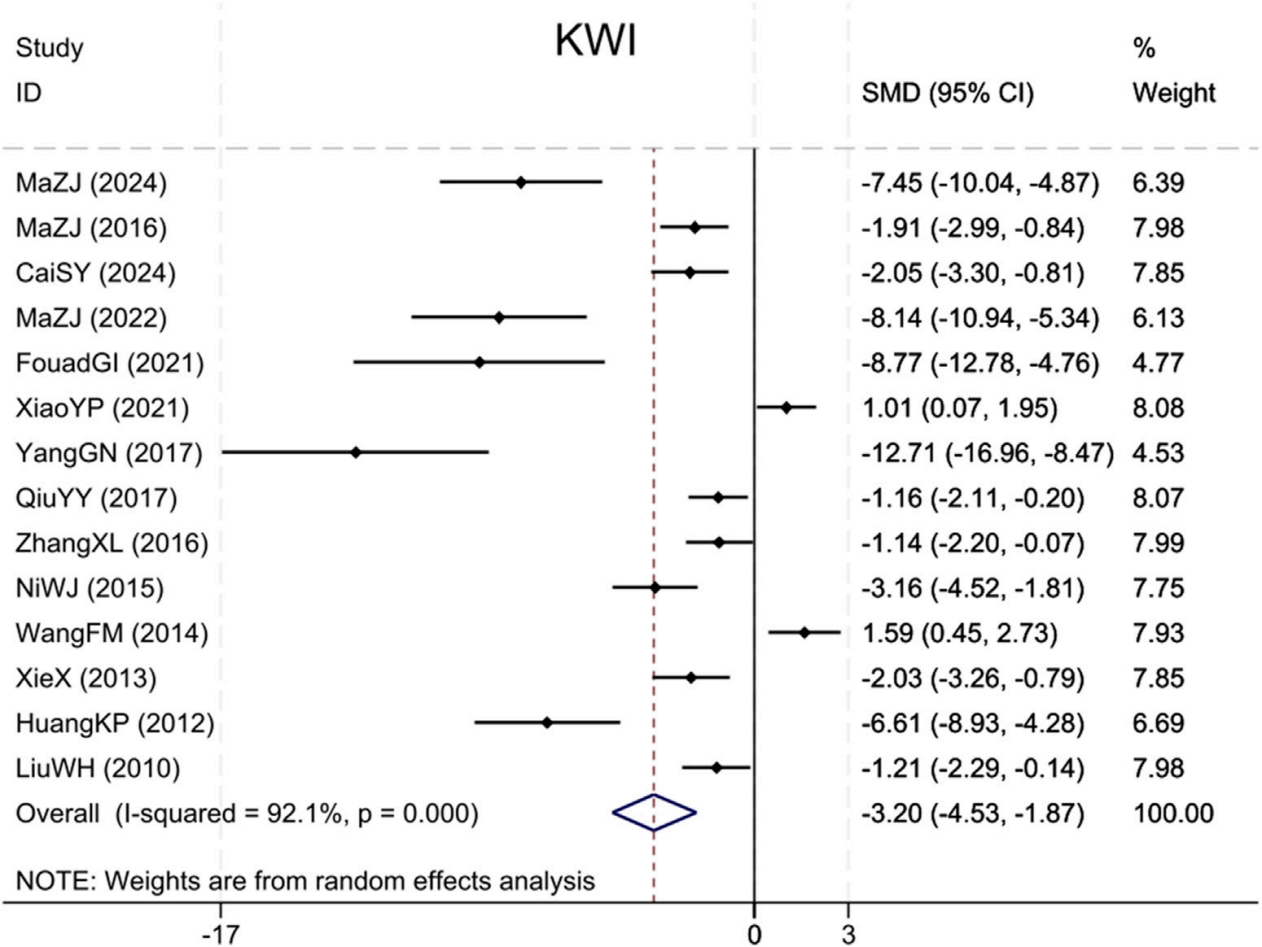
Forest plot for KWI.

Fourteen studies (Liu et al., 2010; Huang et al., 2012; Xie et al., 2013; Wang et al., 2014; Ni et al., 2015; Ma et al., 2016; Zhang et al., 2016; Qiu et al., 2017; Yang et al., 2017; Xiao et al., 2021; Ibrahim Fouad and Ahmed, 2021; Ma et al., 2022; Cai et al., 2024; Ma et al., 2024) cited KWI, with findings indicating that BBR significantly reduced the KWI, versus the model group (Sample size: 252; SMD = −3.20 (95% CI: −4.53 to −1.87), P < 0.001; χ^2^ = 164.46, I^2^ = 92.1%, Figure 7).

#### 3.4.5 Fibrosis markers: collagen I, collagen IV, FN, and E-cadherin

**FIGURE 8:**
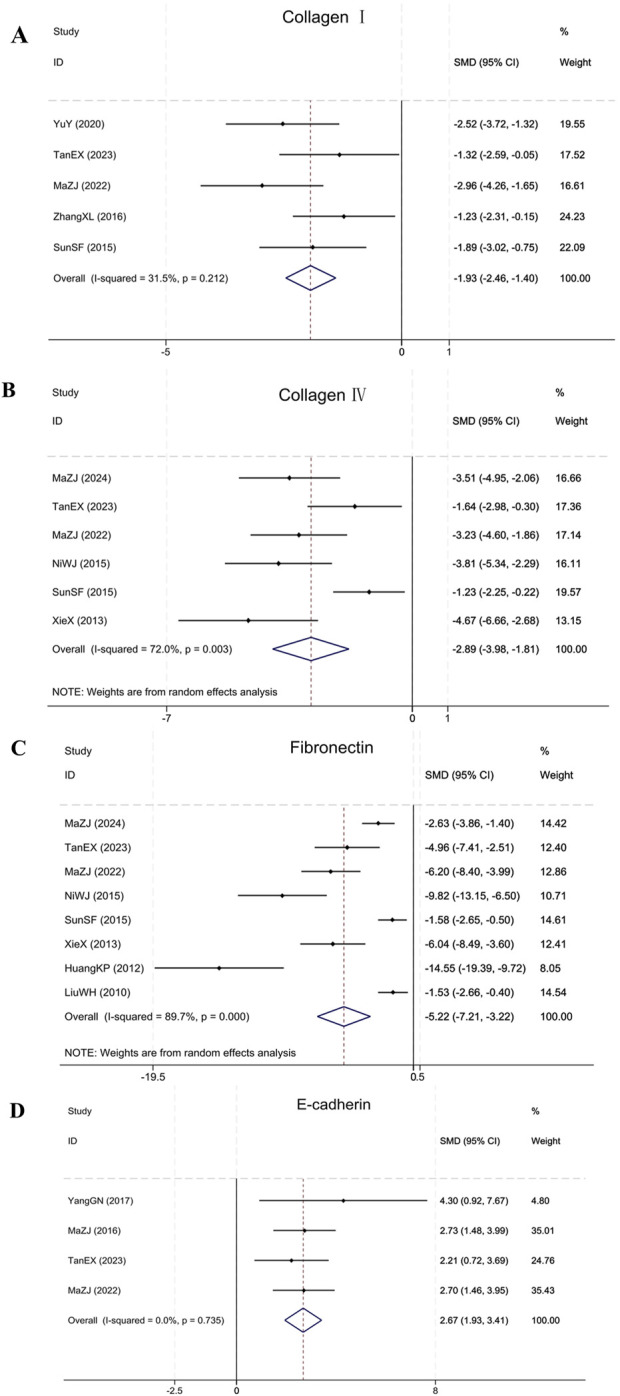
Forest plot for **(A)** Collagen I, **(B)** Collagen IV, **(C)** FN, and **(D)** E-cadherin.

Meta-analysis showed that BBR significantly modulated fibrosis markers in renal fibrosis animals. In the five included studies (Sun et al., 2015; Zhang et al., 2016; Yu et al., 2020; Ma et al., 2022; Tan et al., 2023), BBR significantly reduced Collagen I levels (Sample size: 86; SMD = −1.93 (95% CI: −2.46 to −1.40), P = 0.212; χ^2^ = 5.84, I^2^ = 31.5%). Six studies (Xie et al., 2013; Ni et al., 2015; Sun et al., 2015; Ma et al., 2022; Tan et al., 2023; Ma et al., 2024) showed that BBR was effective in reducing Collagen IV accumulation (Sample size: 106; SMD = −2.89 (95% CI: −3.98 to −1.81), P = 0.003; χ^2^ = 17.83, I^2^ = 72.0%). Eight studies (Liu et al., 2010; Huang et al., 2012; Xie et al., 2013; Ni et al., 2015; Sun et al., 2015; Ma et al., 2022; Tan et al., 2023; Ma et al., 2024) reporting on FN indicated that BBR was effective in reducing FN in comparison to the model group (Sample size: 142; SMD = −5.22 (95% CI: −7.21 to −3.22), P < 0.001; χ^2^ = 68.16, I^2^ = 89.7%). Four studies (Ma et al., 2016; Yang et al., 2017; Ma et al., 2022; Tan et al., 2023) targeting E-cadherin showed that BBR significantly upregulated E-cadherin expression (Sample size: 58; SMD = 2.67 (95% CI: 1.93–3.41), P = 0.735; χ^2^ = 1.28, I^2^ = 0.0%). The results of each indicator are presented in Figure 8.

#### 3.4.6 Renal fibrosis area

**FIGURE 9:**
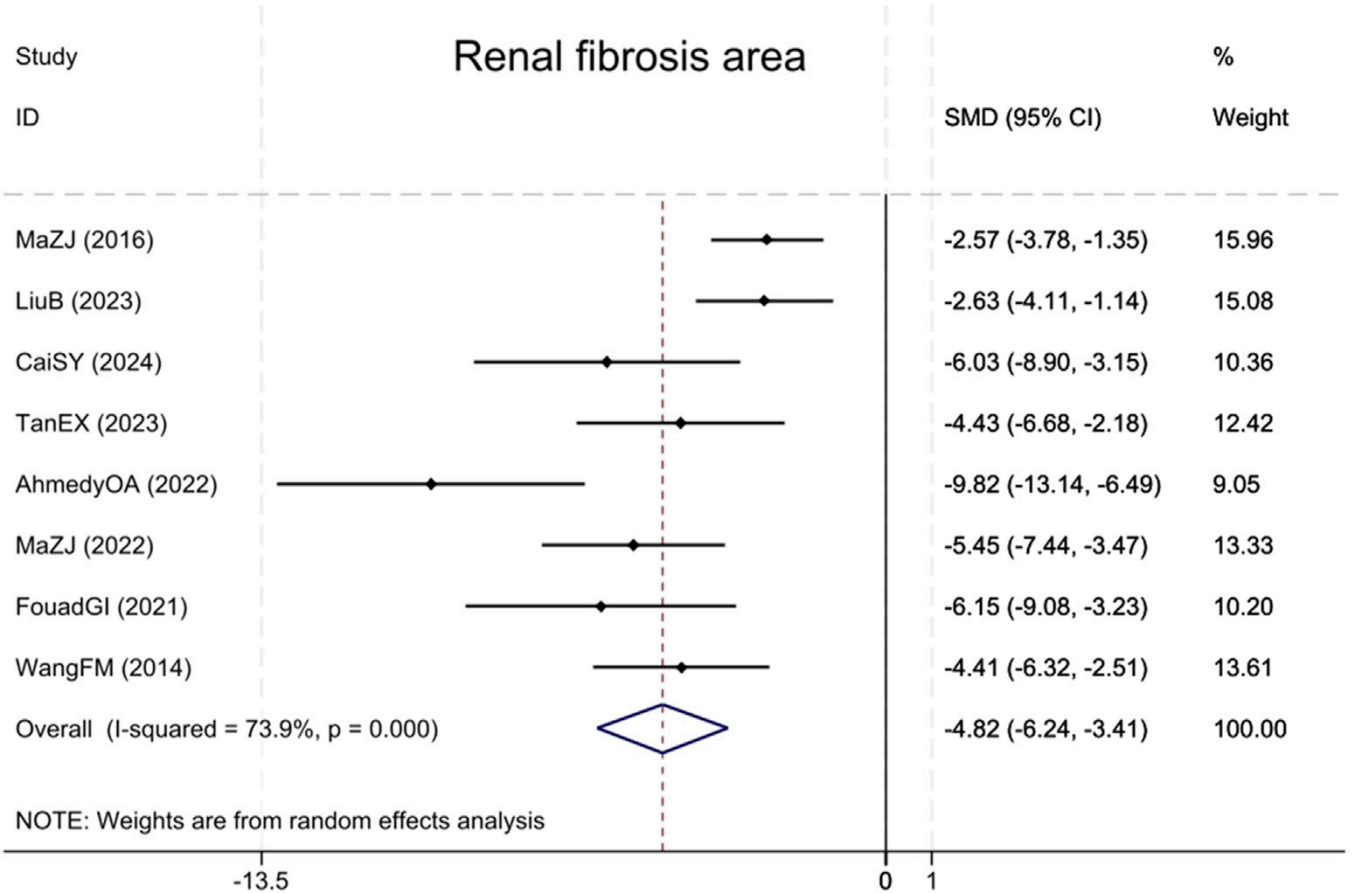
Forest plot for Renal fibrosis area.

Eight studies (Wang et al., 2014; Ma et al., 2016; Ibrahim Fouad and Ahmed, 2021; Ahmedy et al., 2022; Ma et al., 2022; Tan et al., 2023; Liu et al., 2023; Cai et al., 2024) reported the renal fibrotic area, showing that compared with the model group, BBR notably decreased the renal fibrotic area (Sample size: 126; SMD = −4.82 (95% CI: −6.24 to −3.41), P < 0.001; χ^2^ = 26.79, I^2^ = 73.9%, Figure 9).

#### 3.4.7 Renal injury parameters: KIM-1 and kidney injury score

**FIGURE 10:**
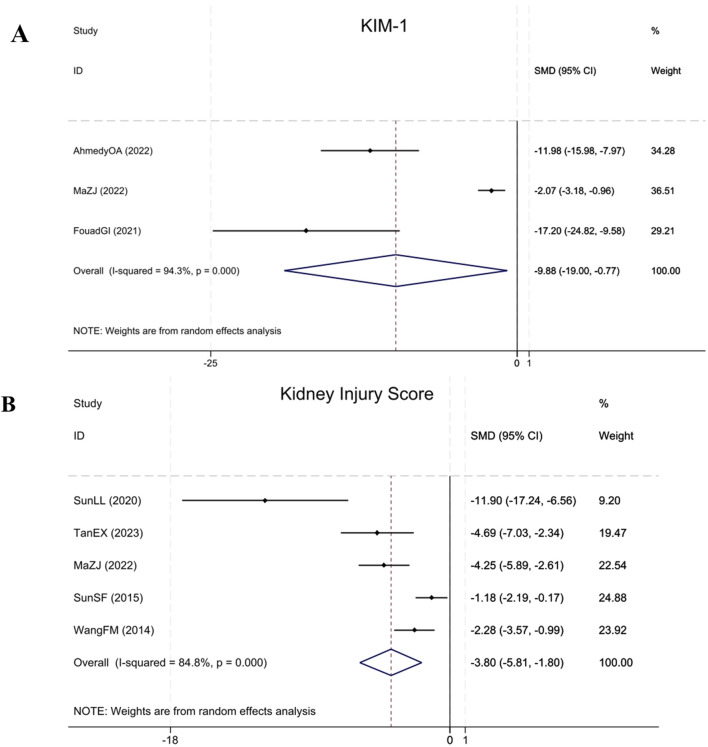
Forest plot for **(A)** KIM-1 and **(B)** Kidney Injury Score.

Three studies (Ibrahim Fouad and Ahmed, 2021; Ahmedy et al., 2022; Ma et al., 2022) cited KIM-1 levels, demonstrating that compared to the model group, BBR significantly reduced KIM-1 in renal fibrosis animals (Sample size: 52; SMD = −9.88 (95% CI: −19.00 to −0.77), P < 0.001; χ^2^ = 35.30, I^2^ = 94.3%). Five studies (Wang et al., 2014; Sun et al., 2015; Sun et al., 2020; Ma et al., 2022; Tan et al., 2023) assessed the Kidney Injury Score, indicating that BBR significantly reduced the Kidney Injury Score in renal fibrosis animals relative to the model group (Sample size: 78; SMD = −3.80 (95% CI: −5.81 to −1.80), P < 0.001; χ^2^ = 26.27, I^2^ = 84.8%). The results of each indicator are elucidated within Figure 10.

#### 3.4.8 Oxidative stress indices: MDA, SOD and GSH-Px

**FIGURE 11:**
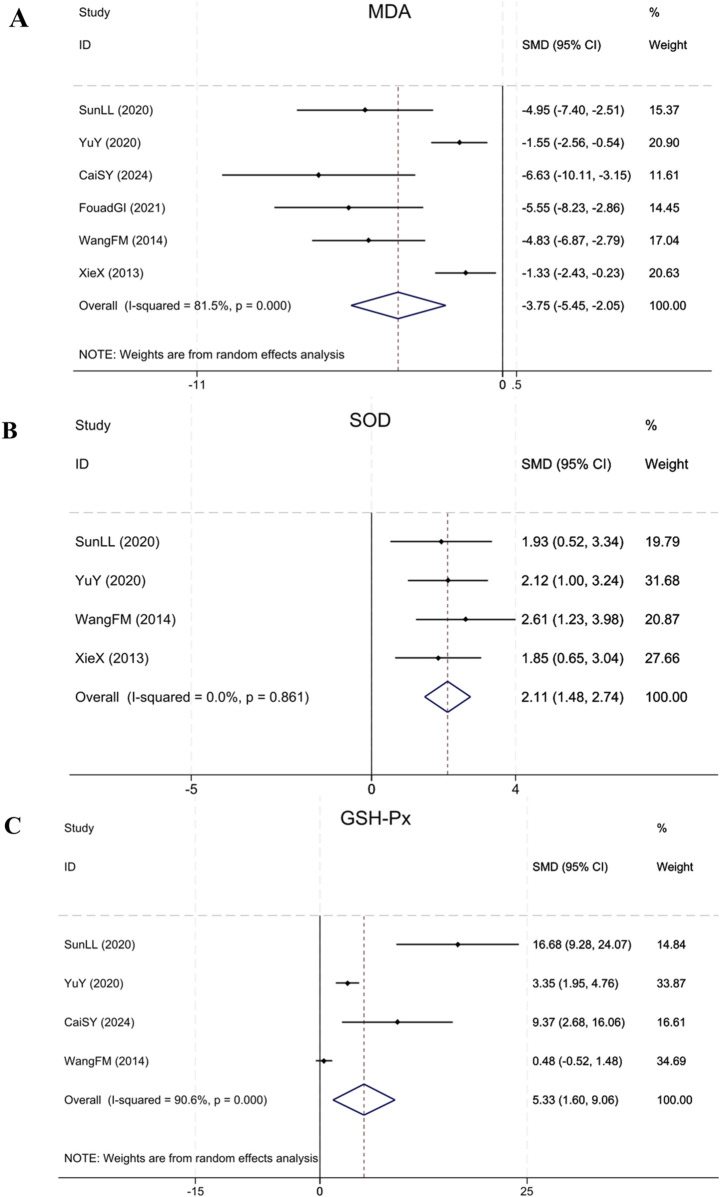
Forest plot for **(A)** MDA, **(B)** SOD and **(C)** GSH-Px.

Six studies (Xie et al., 2013; Wang et al., 2014; Yu et al., 2020; Sun et al., 2020; Ibrahim Fouad and Ahmed, 2021; Cai et al., 2024) reporting MDA showed that in contrast to the model group, BBR notably decreased MDA levels in renal fibrosis animals (Sample size: 86; SMD = −3.75 (95% CI: −5.45 to −2.05), P < 0.001; χ^2^ = 26.95, I^2^ = 81.5%). Four studies (Xie et al., 2013; Wang et al., 2014; Yu et al., 2020; Sun et al., 2020) reporting SOD showed that BBR notably upregulated the expression of SOD in treatment group relative to model group (Sample size: 64; SMD = 2.11 (95% CI: 1.48–2.74), P = 0.861; χ^2^ = 0.75, I^2^ = 0.0%). Four studies (Wang et al., 2014; Yu et al., 2020; Sun et al., 2020; Cai et al., 2024) on GSH-Px found that compared to the model group, BBR significantly upregulated GSH-Px expression (Sample size: 54; SMD = 5.33 (95% CI: 1.60–9.06), P < 0.001; χ^2^ = 31.89, I^2^ = 90.6%). The results of each indicator are shown in Figure 11.

#### 3.4.9 Inflammation levels:IL-1β, NLRP3

**FIGURE 12:**
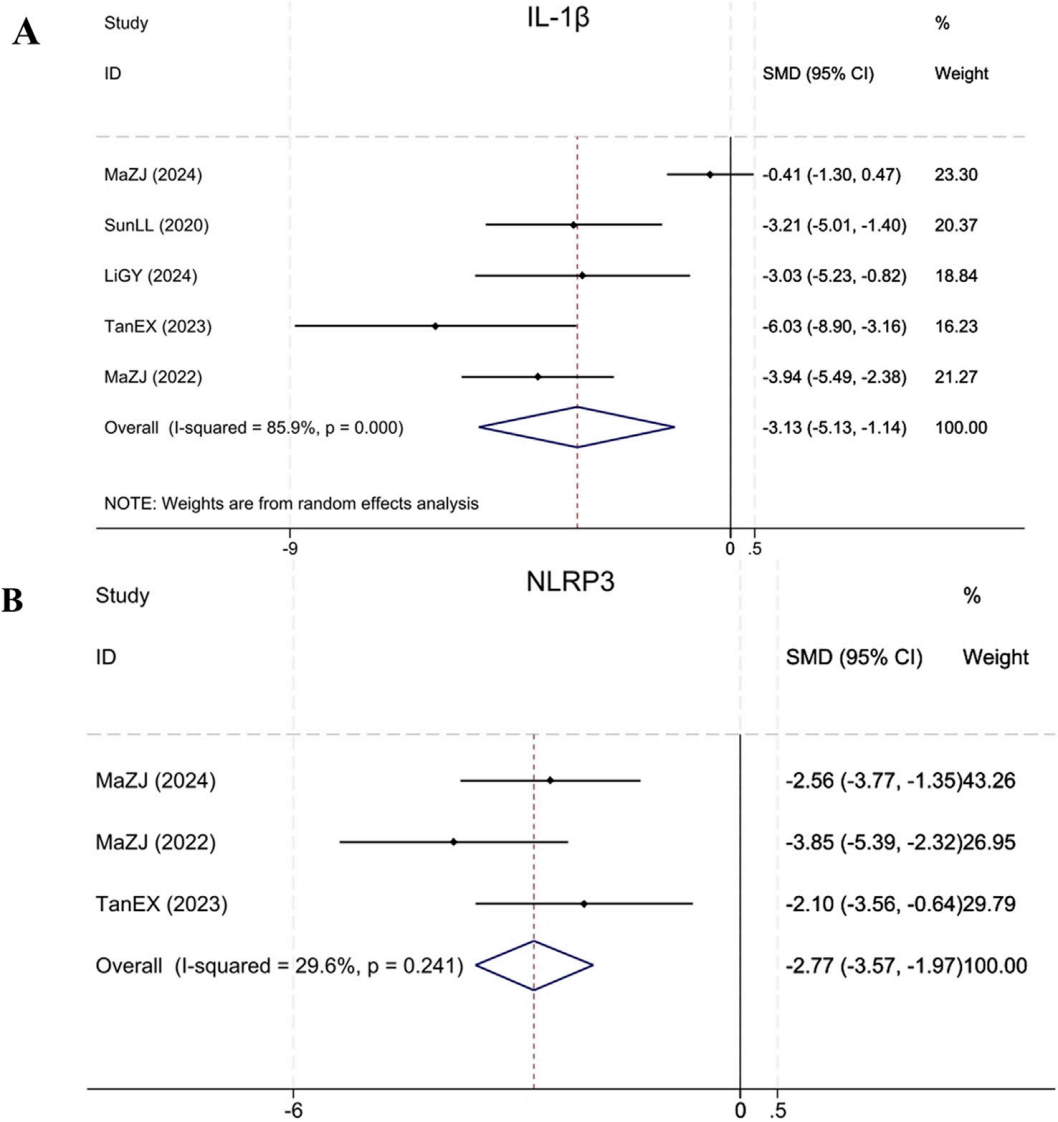
Forest plot for **(A)** IL-1β and **(B)** NLRP3.

Five studies (Sun et al., 2020; Ma et al., 2022; Tan et al., 2023; Ma et al., 2024; Li et al., 2024) reported IL-1β, demonstrating that BBR significantly reduced IL-1β levels in treatment group relative to model group (Sample size: 72; SMD = −3.13 (95% CI: −5.13 to −1.14), P < 0.001; χ^2^ = 28.29, I^2^ = 85.9%). Three studies (Ma et al., 2022; Tan et al., 2023; Ma et al., 2024) reported NLRP3. Compared to the model group, BBR significantly reduced NLRP3 levels in renal fibrosis animals (Sample size: 52; SMD = −2.77 (95% CI: −3.57 to −1.97), P = 0.241; χ^2^ = 2.84, I^2^ = 29.6%). The results of each indicator are shown in Figure 12.

### 3.5 Subgroup analysis

In response to the elevated heterogeneity identified in the included animal experiments, a comprehensive subgroup evaluation was carried out to uncover possible causes of variation. The primary outcome indicators were stratified by publication year, treatment duration, modeling method, animal species, and drug dosages. Results suggest that drug dosages could be the source of heterogeneity for Scr and α-SMA outcomes. Notably in Scr analysis, subgroups receiving ≥200 mg/kg and <200 mg/kg doses both demonstrated reduced heterogeneity. For α-SMA outcomes, the ≥200 mg/kg subgroup exhibited more pronounced heterogeneity reduction than lower dosage groups. Regarding BUN and TGF-β1 outcomes, modeling method appeared to be the predominant source of heterogeneity. Stratification by modeling method revealed substantial heterogeneity reduction within the UUO model subgroup while other classification factors demonstrated limited impact. This result remained consistent across both BUN and TGF-β1 analyses. Supplementary Table S3 presents the detailed result.

### 3.6 Sensitivity analysis

Using the “leave-one-out” method, each outcome indicator was evaluated by excluding individual studies one at a time to evaluate the reliability of the results. The GSH-Px assessment, with the exclusion of the Wang et al. (2014) research, modified the overall effect size, yet the direction of the effect for other indicators was unchanged. Supplementary Appendix Figure S1 depicts the results.

### 3.7 Publication bias

For evaluating publication bias for Scr, BUN, TGF-β1, and 24-h urinary protein, we employed funnel plot analysis and the Egger test. Regarding 24-h urinary protein, the Egger test did not demonstrate statistical significance (P = 0.689), and the corresponding funnel plot exhibited symmetry, indicating minimal bias likelihood. In contrast, for these remaining indicators (Scr, BUN, and TGF-β1), the Egger test all demonstrated P = 0.000 < 0.05, and the corresponding funnel plots showing significant asymmetry. These findings suggest potential publication bias or lower-quality studies due to unpublished negative results or methodological heterogeneity. Supplementary Appendix Figure S2 shows the results.

### 3.8 Berberine: time-dose response analysis

**FIGURE 13:**
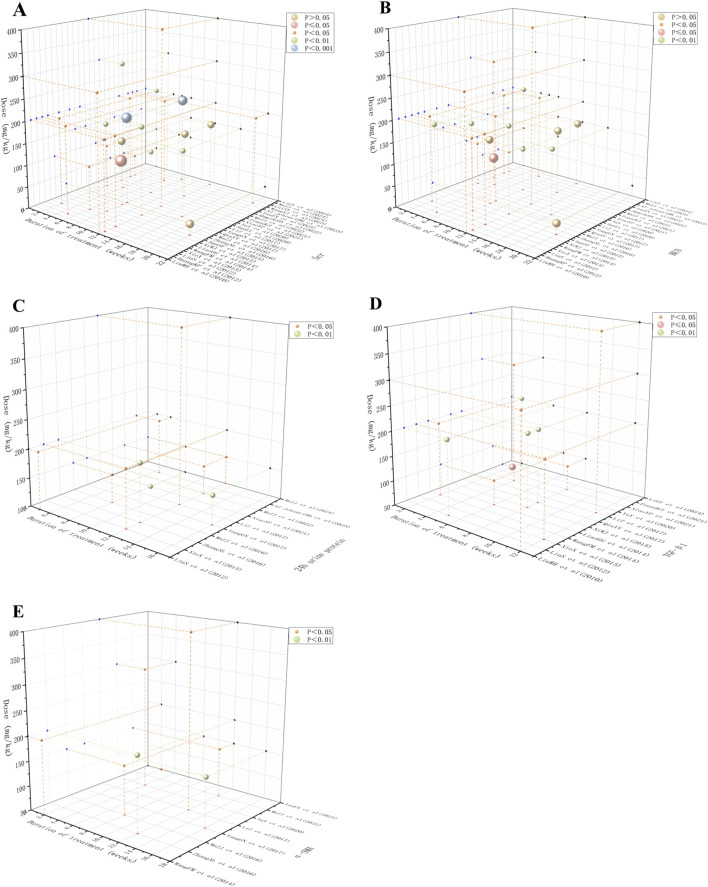
Scatter plot of time-dose-response for **(A)** Scr, **(B)** BUN, **(C)** 24 h urine protein, **(D)** TGF-β1 and **(E)** α-SMA.

Scr and BUN are essential biomarkers for evaluating renal impairment, reflecting glomerular filtration rate and nitrogen metabolism abnormalities (Lopez-Giacoman and Madero, 2015). The 24-h urinary protein level serves as an essential clinical indicator for evaluating glomerular filtration barrier’s structural integrity, with its elevated levels strongly linked to increased glomerular basement membrane permeability and podocyte damage (Zeng et al., 2024). TGF-β1, a key regulator of the pro-fibrotic process, promotes fibroblast proliferation in the renal interstitium and extracellular matrix deposition through Smad pathway activation (Ma and Meng, 2019). α-SMA serves as a definitive indicator of myofibroblast activation, with its expression intensities positively correlating significantly with the severity of renal tubulointerstitial fibrosis (Jercan et al., 2012). Considering the key pathological indicators mentioned above, we systematically explored the time-dose response characteristics of BBR in treating renal fibrosis. Our results revealed that 50 mg/kg was the minimum effective dose for improving Scr and BUN, whereas the highest administered concentration reached 400 mg/kg. The shortest effective treatment period for Scr was 2 weeks, with the longest being 20 weeks, while for BUN, it varied between 2 and 16 weeks. The therapeutic dosage range for α-SMA and TGF-β1 was also 50–400 mg/kg, with α-SMA showing an effective treatment period of 2 weeks at the shortest and 16 weeks at the longest, and TGF-β1 being effective from 2 to 12 weeks. The effective dose for 24-h urinary protein varied between 100 mg/kg and 400 mg/kg, with an effective treatment duration of 5–16 weeks. In conclusion, BBR can effectively improve renal function, reduce proteinuria, and inhibit fibrosis with optimal efficacy achieved within a dose range of 100–400 mg/kg for 5–12 weeks of treatment. These findings are illustrated in Figure 13.

## 4 Discussion

### 4.1 Evidence synthesis

We systematically assessed the time-dose response associations and the specificity of action mechanisms of berberine (BBR) across diverse renal fibrosis models by synthesizing data from 26 pre-clinical studies. The meta-analysis revealed that BBR significantly improved renal function, reduced proteinuria, mitigated renal injury, inhibited fibrosis, and attenuated oxidative stress and inflammation. In comparison to previous studies that primarily focused on single models, our multi-model synthesis offers a more comprehensive perspective on BBR’s efficacy across different fibrotic conditions. This approach enhances the understanding of BBR’s multifaceted mechanisms, supporting its broader therapeutic potential. Unlike current therapies, BBR’s multi-targeted suppression of TGF-β1/Smad signaling, oxidative stress, and inflammatory mediators may address the multifactorial nature of fibrosis, potentially overcoming therapeutic resistance in monotherapy approaches.

The time-dose-response analysis indicated that the optimal efficacy of BBR was observed at doses between 100–400 mg/kg and over a therapeutic period of 5–12 weeks. Subgroup analyses of primary outcome metrics suggested that different model types and administered doses were the main sources of inter-study heterogeneity. These findings align with prior research, which indicates that variability in fibrotic responses can result from differing experimental models and compound administration (Wynn, 2008). Additionally, another study have emphasized the importance of considering model specific responses to optimize treatment methods (Rockey et al., 2015). These insights underscore the necessity of customizing BBR applications to meet specific experimental needs. Furthermore, sensitivity analyses demonstrated that the direction of effect of most outcome indicators was insensitive to single-study exclusion, supporting the robustness of the results, except for the GSH-Px indicator, where the effect size was significantly altered after the exclusion (Wang et al., 2014) of this study. However, the publication bias assessment revealed significant asymmetry in the funnel plots for Scr, BUN, and TGF-β1, suggesting potential unpublished negative results or methodological heterogeneity, while the risk of publication bias was low for 24 h urine protein. Taken together, these findings indicate that while BBR shows significant potential for anti-renal fibrosis across different models, the small sample size and high heterogeneity highlight the need for further studies to validate the clinical relevance of these heterogeneous metrics and to refine experimental designs to minimize bias.

### 4.2 Mechanism-specific analysis of berberine against renal fibrosis in the included literature

#### 4.2.1 UUO model

BBR demonstrates multi-target therapeutic effects against renal fibrosis induced by UUO. Research evidence indicates that BBR can suppress the NLRP3 inflammasome activation, decrease IL-1β levels, and reduce inflammatory cell infiltration (Tan et al., 2023; Wang et al., 2014). Its anti-apoptotic effect involves modulating the balance between Bcl-2 and Bax proteins and suppressing caspase-3 activation (Tan et al., 2023). Furthermore, BBR can activate the AMP-activated protein kinase (AMPK) signaling pathway, promoting fatty acid oxidation (FAO) through upregulation of PPARα and CPT1A expression, which subsequently ameliorates energy metabolism in renal tubular epithelial cells and reverses epithelial-mesenchymal transition (EMT) phenotypes (Tan et al., 2023). BBR’s antioxidant effects are manifested through increased activities of SOD and catalase (CAT), reduced MDA levels, and modulation of the TGF-β1/Smad3 pathway to downregulate α-SMA expression and inhibit myofibroblast accumulation (Wang et al., 2014; Liu et al., 2023). Notably, BBR induces ferroptosis in renal myofibroblasts by decreasing Fe^2+^, MDA, and reactive oxygen species (ROS) levels, while modulating the expression of glutathione peroxidase 4 (GPX4) and acyl-CoA synthetase long-chain family member 4 (ACSL4) proteins (Liu et al., 2023). Additionally, BBR can improve renal hemodynamics and downregulate the NF-κB p65/TGF-β1/CTGF pathway, jointly suppressing the pathological advancement of fibrotic processes (Li et al., 2024).

#### 4.2.2 Diabetic nephropathy models

BBR alleviates renal fibrosis in diabetic kidney disease via multi-target synergistic mechanisms, including metabolic regulation, inhibition of inflammatory and fibrotic signaling pathways, antagonism of EMT, and renal structural protection.

##### 4.2.2.1 Regulation of glucose-lipid metabolism and antioxidant effects

In DN animal models, BBR shows notable hypoglycemic effectiveness through insulin sensitivity enhancement and insulin secretion potentiation. Concurrently, It can alleviate lipid metabolism disorders by decreasing cholesterol, low-density lipoprotein cholesterol (LDL-C), and serum triglycerides. In terms of Its antioxidant effects, BBR activates the nuclear factor erythroid 2-related factor 2 signaling pathway and upregulates HO-1 and NQO1 expression. These actions work in tandem to enhance the endogenous antioxidant capacity and reduce the accumulation of MDA and ROS. Moreover, BBR exerts regulatory effects on proteins linked to iron homeostasis, such as divalent metal transporter 1 and transferrin receptor. By modulating their expression, BBR alleviates iron overload, suppresses ferroptosis, and preserves the structural and functional wholeness of mitochondria (Al-Jebouri and Al-Murshidi, 2023; Zhang et al., 2016; Cai et al., 2024; Wang et al., 2023).

##### 4.2.2.2 Suppression of inflammation and NF-κB pathway

BBR exerts a substantial impact in curbing inflammation by regulating the NF-κB pathway. This therapeutic pathway not only reduces the production of inflammatory mediators like MCP-1, IL-1β, and TNF-α, but also inhibits the influx of monocytes and macrophages into kidney. Moreover, BBR inhibits AGEs from attaching to their receptor, RAGE, which disrupts the AGEs-RAGE-TGF-β/Smad2 signaling cascade and reduces the phosphorylation of protein kinase C-β (PKC-β), thereby further mitigating inflammation. In addition to this, BBR can downregulate NLRP3 inflammasome activity, inhibiting the discharge of cleaved caspase-1 and IL-1β. These combined effects notably reduce inflammation-induced renal injury (Liu et al., 2010; Sun et al., 2015; Qiu et al., 2017; Ma et al., 2022).

##### 4.2.2.3 Modulation of fibrosis-related signaling pathways

BBR inhibits abnormal ECM deposition by targeting multiple pro-fibrotic signaling pathways. It significantly suppresses the TGF-β1/Smad pathway, reducing expression of phosphorylated Smad2/3 and connective tissue growth factor (CTGF), while upregulating Smad7 and the nuclear transcriptional repressor SnoN to restore TGF-β1/SnoN homeostasis. Additionally, berberine synergistically reduces renal fibrosis by suppressing the Notch/Snail pathway (downregulating Jagged1, Notch1, and Snail1) and the RhoA/ROCK pathway (reducing FN accumulation), as well as blocking the SphK1- S1P signaling cascade (decreasing S1P2 receptor expression) (Liu et al., 2012; Yang et al., 2017; Xie et al., 2013; Huang et al., 2012).

##### 4.2.2.4 Anti-epithelial-mesenchymal transition and cytoprotection

BBR effectively inhibits EMT triggered by high glucose levels in renal tubular epithelial cells. This is achieved by upping the production of E-cadherin and decreasing levels of α-SMA and vimentin. It also reestablishes the balance between MMPs and TIMPs, thereby reducing deposition of type IV collagen and FN. Furthermore, BBR modulates the methylation status of the KLF4 promoter by inhibiting DNA methyltransferases and enhancing the expression of DNA demethylases, thus restoring the renoprotective function of KLF4 (Yang et al., 2017; Ni et al., 2015; Cai et al., 2024).

##### 4.2.2.5 Improvement of renal pathology and function

BBR significantly ameliorates renal histopathological damage, particularly manifested by hypertrophic glomeruli, expanded mesangial matrices, thickened basement membranes, and interstitial fibrosis. Concurrently, BBR effectively reduces urinary protein excretion and improves glomerular filtration barrier function. Furthermore, by suppressing glomerular endothelial cell pyroptosis and maintaining the structural integrity of renal tubular epithelial cells, BBR can delays renal function decline (Al-Jebouri and Al-Murshidi, 2023; Sun et al., 2015; Ma et al., 2024).

#### 4.2.3 Drug-induced models

In cisplatin-induced kidney fibrosis models, BBR can downregulate tumor necrosis factor-α (TNF-α) to attenuate inflammatory responses. At the same time, it targets the P2X7R/DUSP6/ERK1/2 and SIRT2/MDM2 pathways. This effectively reduces key biomarkers like KIM-1, galectin-3, and α-SMA, and alleviates kidney impairment (Ahmedy et al., 2022). In doxorubicin-induced renal fibrosis models, BBR reduces collagen deposition and apoptosis by inhibiting the NF-κB/TGF-β1 signaling pathway and increasing antioxidant enzymes’ efficacy, including SOD and CAT (Ibrahim Fouad and Ahmed, 2021). As for the adenine-induced renal fibrosis models, BBR can modulate the PTEN/PI3K/AKT signaling pathway, suppressing TGF-β1 and α-SMA expression while improving antioxidant defense mechanisms through elevated GSH-Px and SOD activities and reduced MDA levels, thereby restoring redox equilibrium (Yu et al., 2020).

#### 4.2.4 Renal ischemia-reperfusion (I/R) model

In I/R models, BBR stimulates the AMPK pathway for lipid metabolism regulation, upregulates GPX4 and ACSL4, thus preventing lipid peroxidation. At the same time, it reduces IL-1β, TNF-α, and MDA, alleviating inflammation and oxidative stress-induced renal damage (Sun et al., 2020).

#### 4.2.5 Unilateral renal artery stenosis model

In unilateral renal artery stenosis models included in the study, BBR attenuates renal interstitial fibrosis and tissue remodeling by deregulating TGF-β1 expression and improving renal function (Miao and Zhang, 2014).

#### 4.2.6 Core antifibrotic mechanisms and model-specific adaptations

**FIGURE 14:**
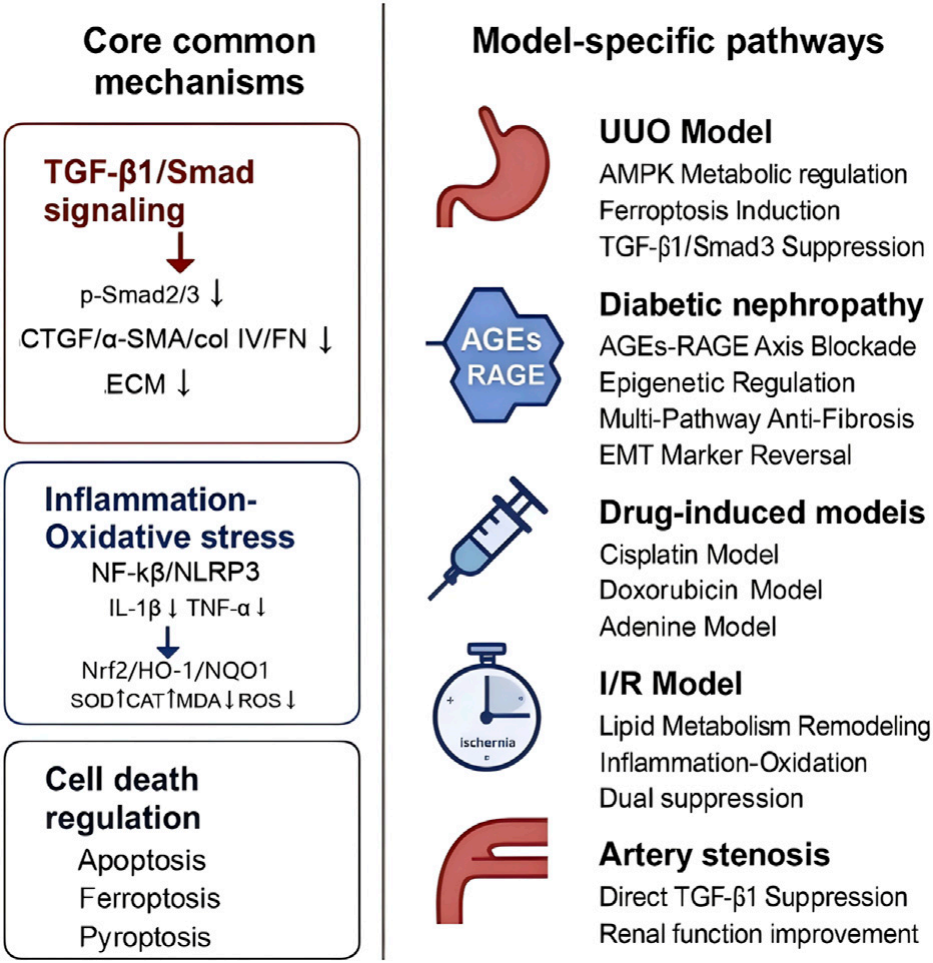
Schematic illustration of berberine’s antifibrotic mechanisms across renal fibrosis models.

The above analysis indicates that BBR addresses renal fibrosis across diverse models through three core mechanisms: (1) TGF-β1/Smad signaling suppression through inhibition of phosphorylated Smad2/3 and downstream profibrotic markers (α-SMA, CTGF, collagen IV, FN), thereby attenuating ECM deposition; (2) inflammation-oxidative stress axis antagonism by downregulating NF-κB/NLRP3 inflammasome activity (reducing IL-1β, TNF-α) while restoring redox homeostasis via Nrf2/HO-1/NQO1 activation, SOD/CAT upregulation, and ROS/MDA suppression; and (3) regulation of cell death pathways, including apoptosis (Bcl-2/Bax/caspase-3 modulation), ferroptosis (GPX4/ACSL4 adjustment), and pyroptosis inhibition. The schematic illustration are provided in Figure 14.

In obstructive nephropathy (UUO models), BBR predominantly addresses ischemia-induced tubular metabolic dysfunction by restoring FAO via AMPK/PPARα activation, while selectively inducing ferroptosis in ECM-producing myofibroblasts through Fe^2+^/MDA/ROS reduction. For diabetic nephropathy, BBR primarily focuses on regulating both metabolic homeostasis and epigenetic modifications. It improves systemic glucolipid disturbances by enhancing insulin sensitivity and reducing LDL-C/triglyceride levels, while also modulating the methylation status of the KLF4 promoter via DNMT/TET to reprogram gene expression. In toxin/hypoxia-driven models (cisplatin/adenine/I/R), acute cytoprotection predominates through rapid redox rebalancing (GSH-Px/SOD activation) and injury-specific pathways, such as KIM-1/galectin-3 suppression and PTEN/PI3K/AKT regulation.

However, studies on drug-induced (e.g., cisplatin/adenine), renal artery stenosis, and renal ischemia-reperfusion (I/R) fibrosis models remain markedly limited, which may introduce bias into mechanistic interpretations. Despite supplementary investigations, data for these models are scarce, hindering comprehensive cross-model integration of mechanisms. Consequently, these findings necessitate further validation through larger sample sizes and more diverse experimental designs.

### 4.3 Limitations of the study and future research directions

This research presents a comprehensive assessment of the efficacy and underlying mechanisms of BBR in combating renal fibrosis by integrating multi-model data. However, several limitations must be acknowledged. 1) the restricted quantity of studies incorporated and their heterogeneous methodological quality—particularly due to insufficient detailed information regarding randomization methods, allocation concealment, and blinding details—may introduce selection and performance biases, thus compromising the reliability of the results. 2) the high inter-study heterogeneity restricts the generalizability of the efficacy assessments. This heterogeneity primarily stems from drug dosages and modeling method. Therefore, efficacy extrapolation to lower-dose regimens or different disease models requires caution. 3) funnel plot asymmetry for key indicators suggests potential publication bias, as unpublished data or the lack of negative results may overestimate the therapeutic effects of BBR. 4) clinical translational evidence is scarce because current conclusions are based entirely on animal studies. This necessitates further validation of BBR’s effectiveness and safety through clinical trials.

Future research should prioritize integrating multi-omics technologies, such as metabolomics and single-cell sequencing, to comprehensively investigate BBR’s target networks across various pathological stages of renal fibrosis and to explore its interaction with the gut microbiota. Emerging evidence (Xu et al., 2022) suggests that changes in specific microbial taxa (e.g., Bifidobacterium, *Lactobacillus*) are closely linked to kidney disease progression. Furthermore, BBR has shown potential to ameliorate metabolic disorders and mitigate diverse pathologies through gut microbiota modulation (Wang et al., 2022; Yang et al., 2023). All those findings indicate that BBR may modulate renal fibrosis-related inflammation and metabolic imbalances through metabolites produced by the microbiota. Subsequent studies could establish a “BBR-microbiota-renal fibrosis” tripartite causal model to clarify its regulatory pathways along the gut-kidney axis. Furthermore, current research predominantly focuses on diabetic nephropathy models. Future studies should broaden their scope to include non-diabetic renal fibrosis models (e.g., UUO, ischemia-reperfusion) to validate the generalizability of BBR’s anti-fibrotic mechanisms and provide comprehensive experimental support for precision therapeutic strategies.

## 5 Conclusion

BBR significantly alleviates renal fibrosis in various experimental models through core mechanisms, including the modulation of inflammatory, oxidative stress-related, and profibrotic signaling pathways. While preclinical evidence supports its potential for clinical translation, further high-quality animal studies and clinical trials are essential for rigorously validating its therapeutic profile.

## Data Availability

The raw data supporting the conclusions of this article will be made available by the authors, without undue reservation.

## Glossary

ACSL4: Acyl-CoA synthetase long-chain family member 4
AGEs: advanced glycation end products
BBR: Berberine
BUN: Blood urea nitrogen
CAT: Catalase
CKD: Chronic kidney disease
DN: Diabetic nephropathy
ECM: Extracellular matrix
EMT: Epithelial-mesenchymal transition
FN: Fibronectin
GPX4: Glutathione peroxidase 4
GSH-Px: Glutathione peroxidase
HO-1: heme oxygenase-1
IL-1β: Interleukin-1β
I/R: Ischemia-reperfusion
KIM-1: Kidney injury molecule-1
KWI: Kidney weight index
MCP-1: Monocyte Chemotactic Protein 1
MDA: Malondialdehyde
MMPs: matrix metalloproteinases
MRA: Mineralocorticoid receptor antagonists
NF-κB: nuclear factor kappa-B
NLRP3: NOD-like receptor protein 3
NQO1: NAD(P)H quinone oxidoreductase 1
PAS: Periodic Acid-Schiff
RAGE: receptor for advanced glycation end-products
RAS: Renin-angiotensin system
ROS: Reactive oxygen species
Scr: Serum creatinine
SOD: Superoxide dismutase
SphK1- S1P: sphingosine kinase 1 -sphingosine-1-phosphate
STZ: Streptozotocin
TGF-β1: Transforming growth factor-β1
TIMPs: tissue inhibitors of metalloproteinases
TNF-α: Tumor necrosis factor-α
UUO: Unilateral ureteral obstruction
α-SMA: α-smooth muscle actin
