# Supplementary material for: Time-dose response and mechanistic specificity of berberine in renal fibrosis from a multi-model integration perspective: a systematic review and meta-analysis on animal models
Source: Front Pharmacol. 2025 Jun 11;16:1600408. doi: 10.3389/fphar.2025.1600408 (PMC12187843; doi:10.3389/fphar.2025.1600408)
Supplement: Supplementary file 1 [file DataSheet1.docx]

Supplementary Material

# Supplementary Figures

**Appendix Figure 1.** Sensitivity analysis results for each indicator

| Scr **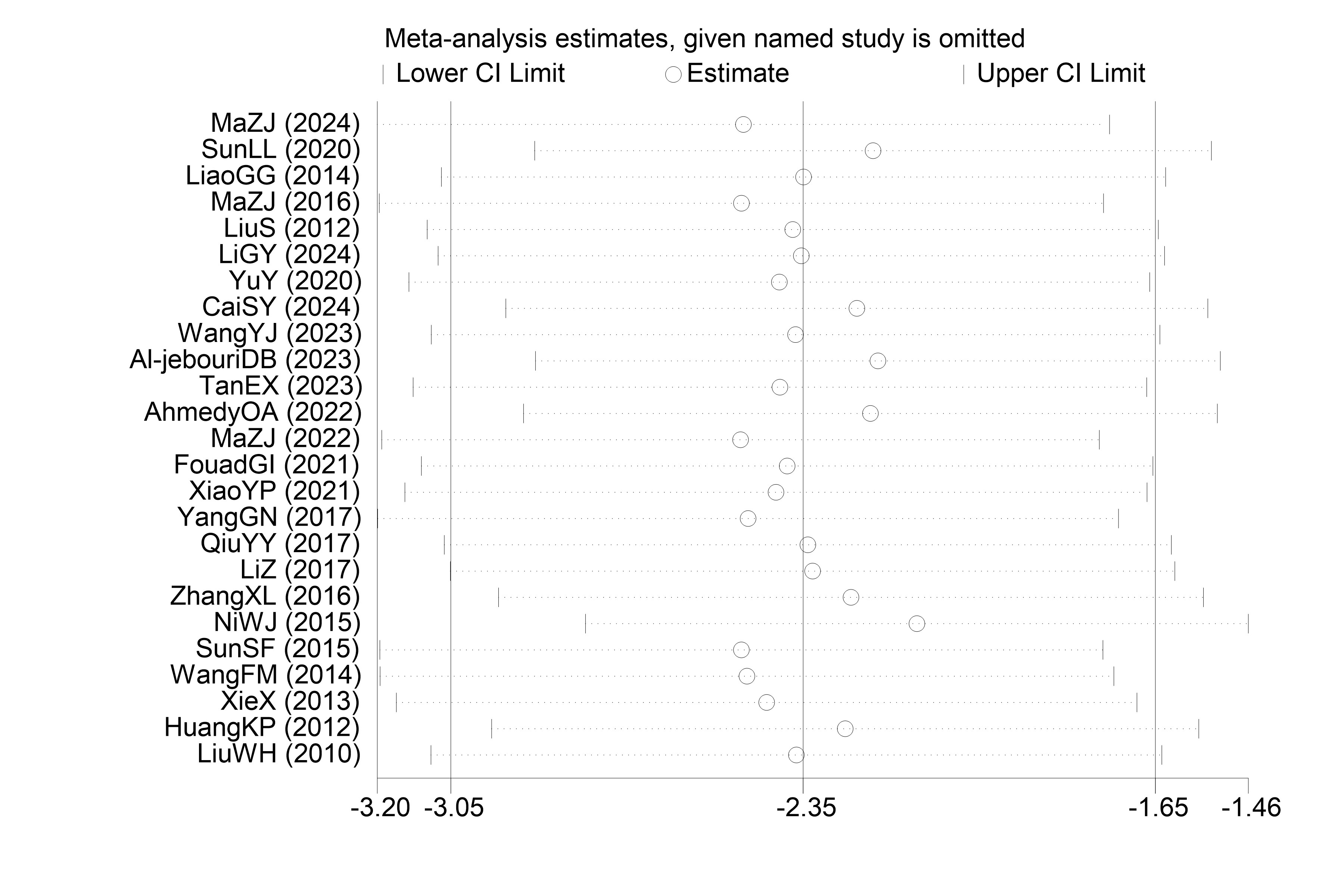** | BUN**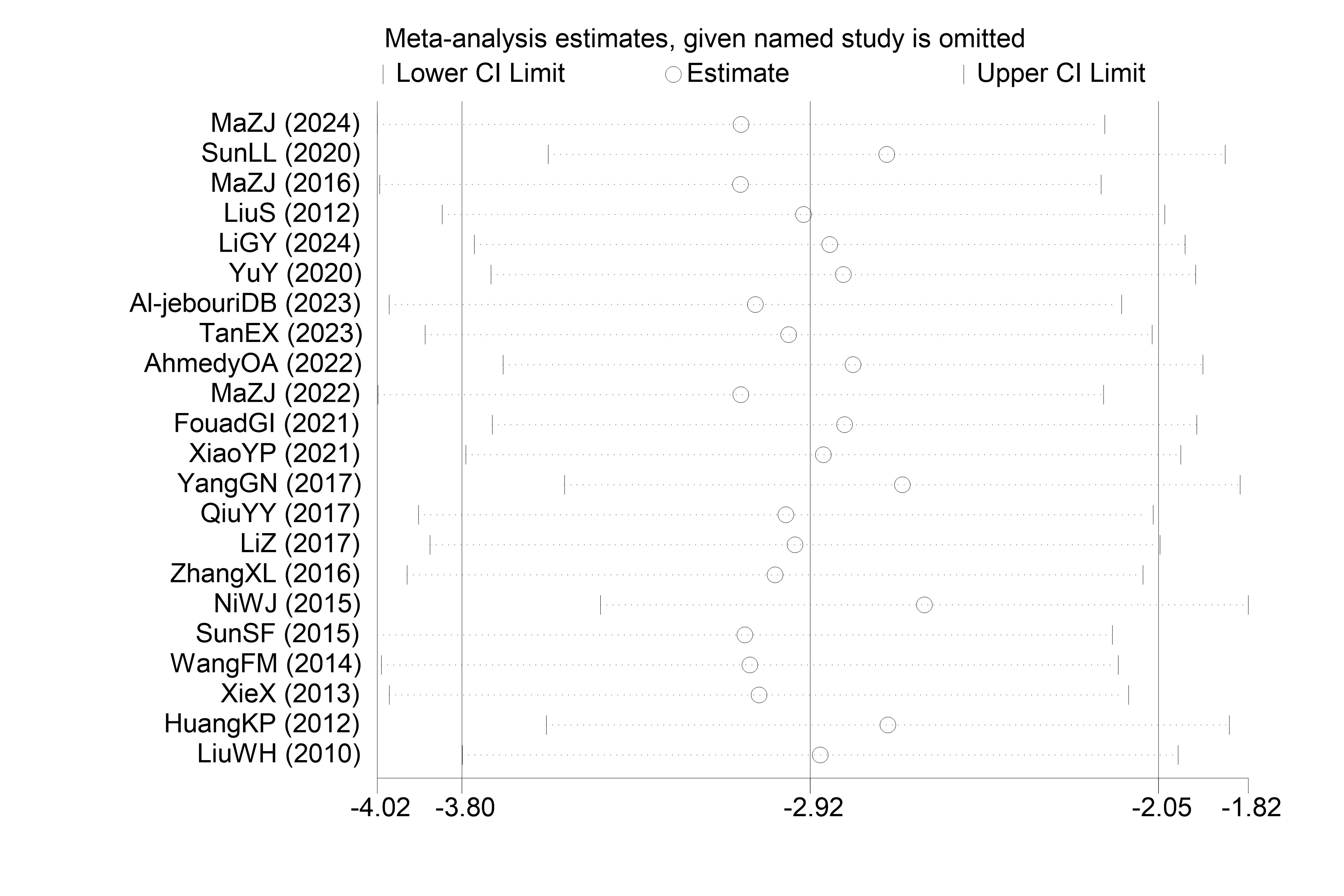** |
| --- | --- |
| TGF-β1 **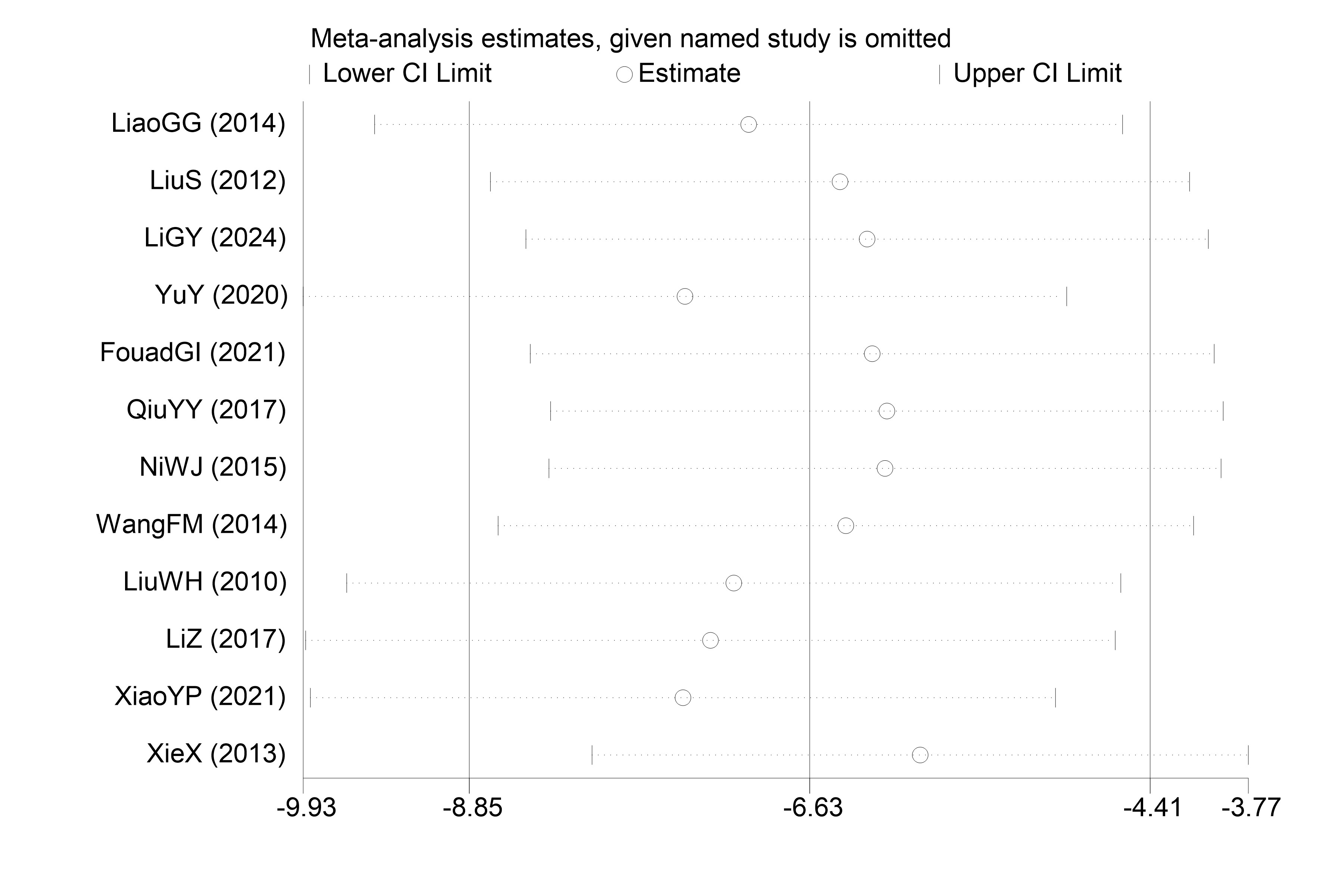** | α-SMA **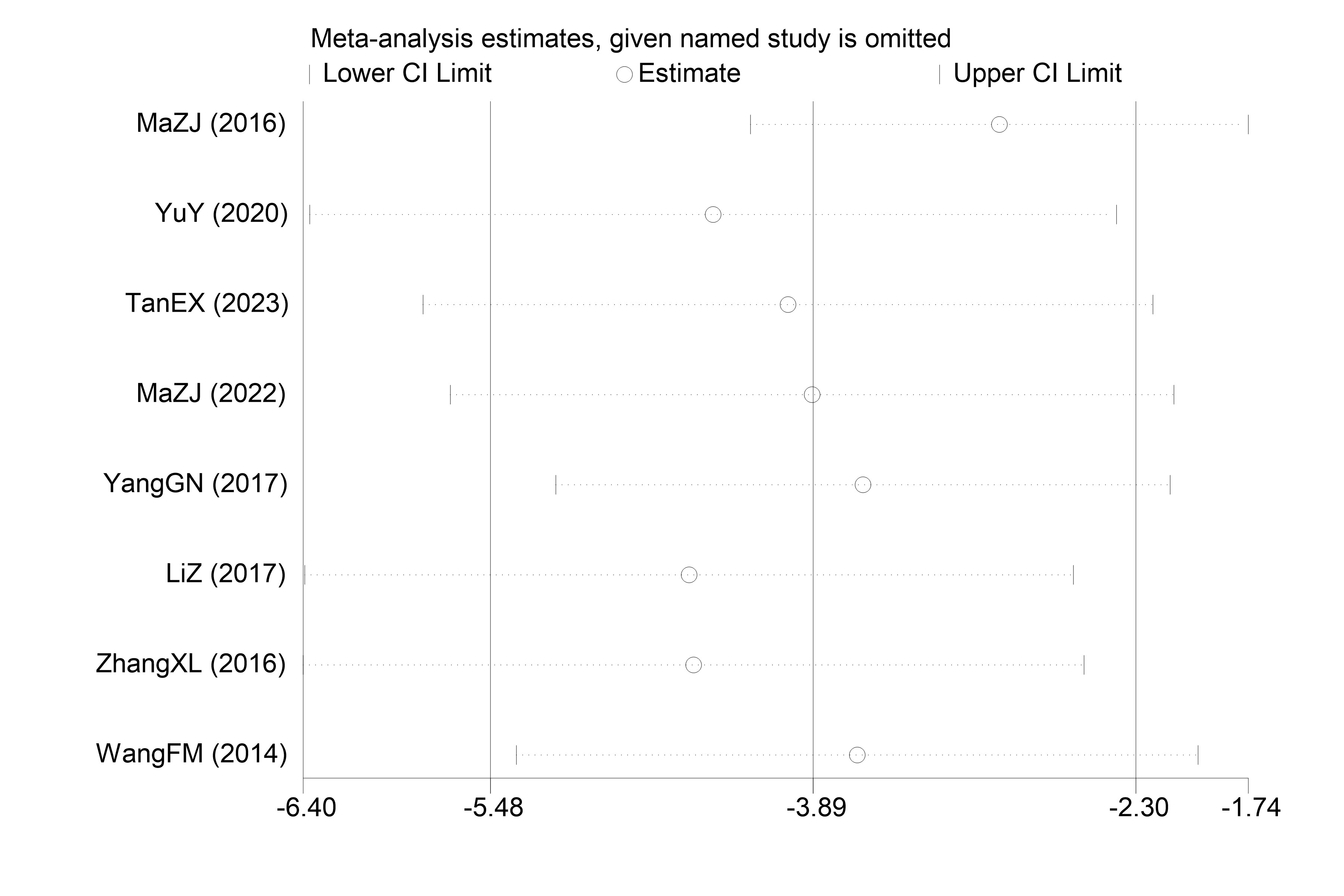** |
| 24h-urine-protein**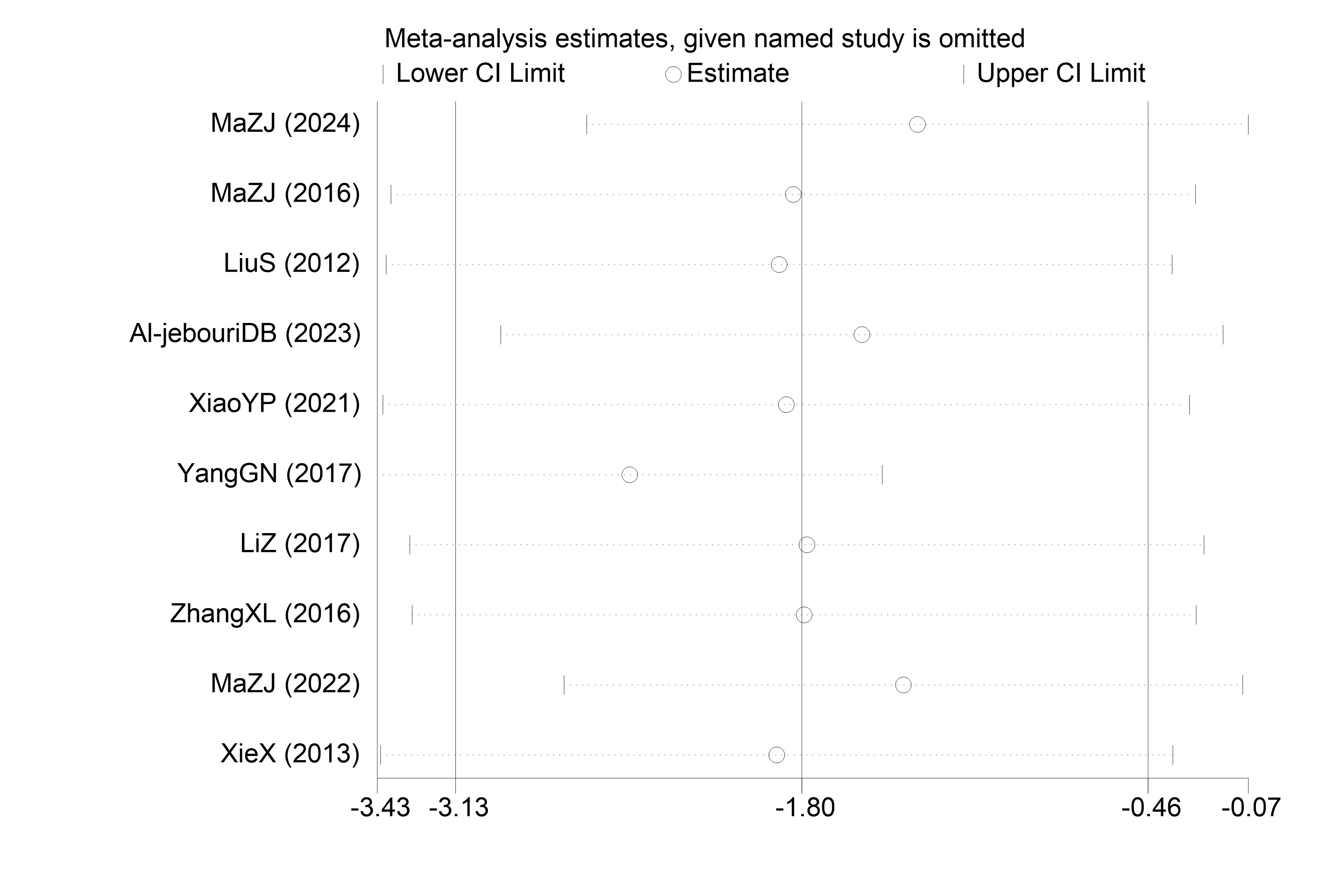** | KWI **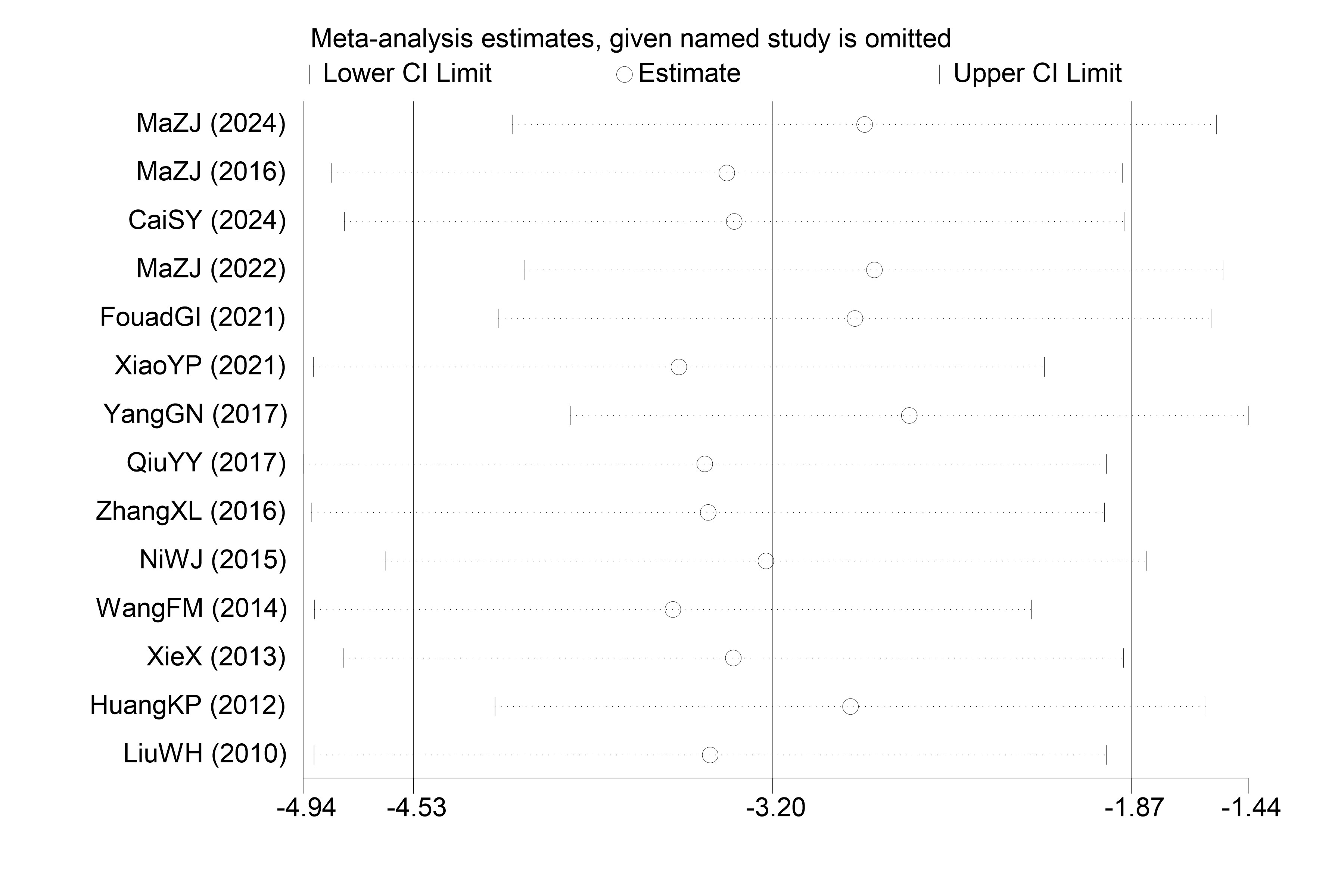** |
| CollagenⅠ**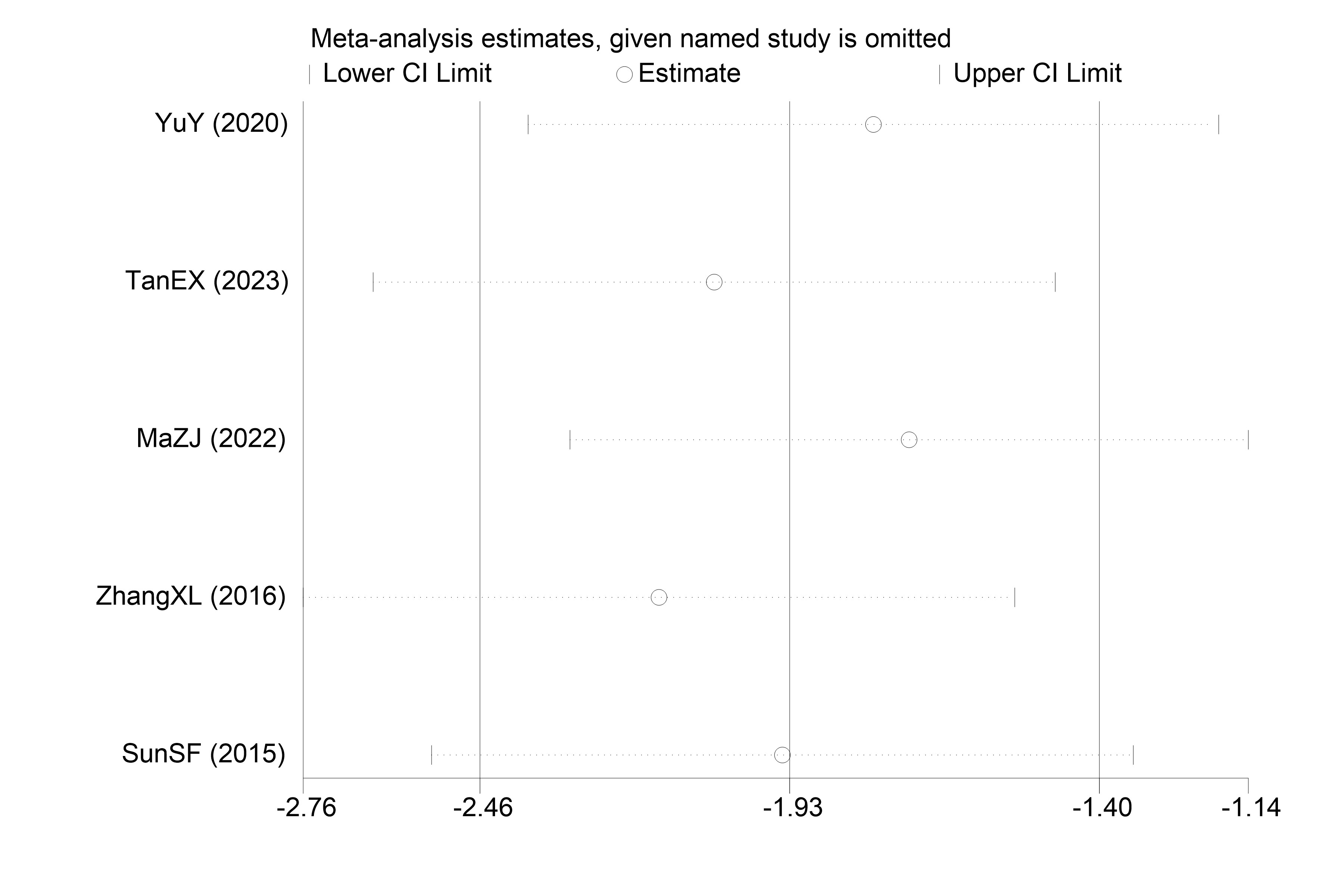** | CollagenⅣ**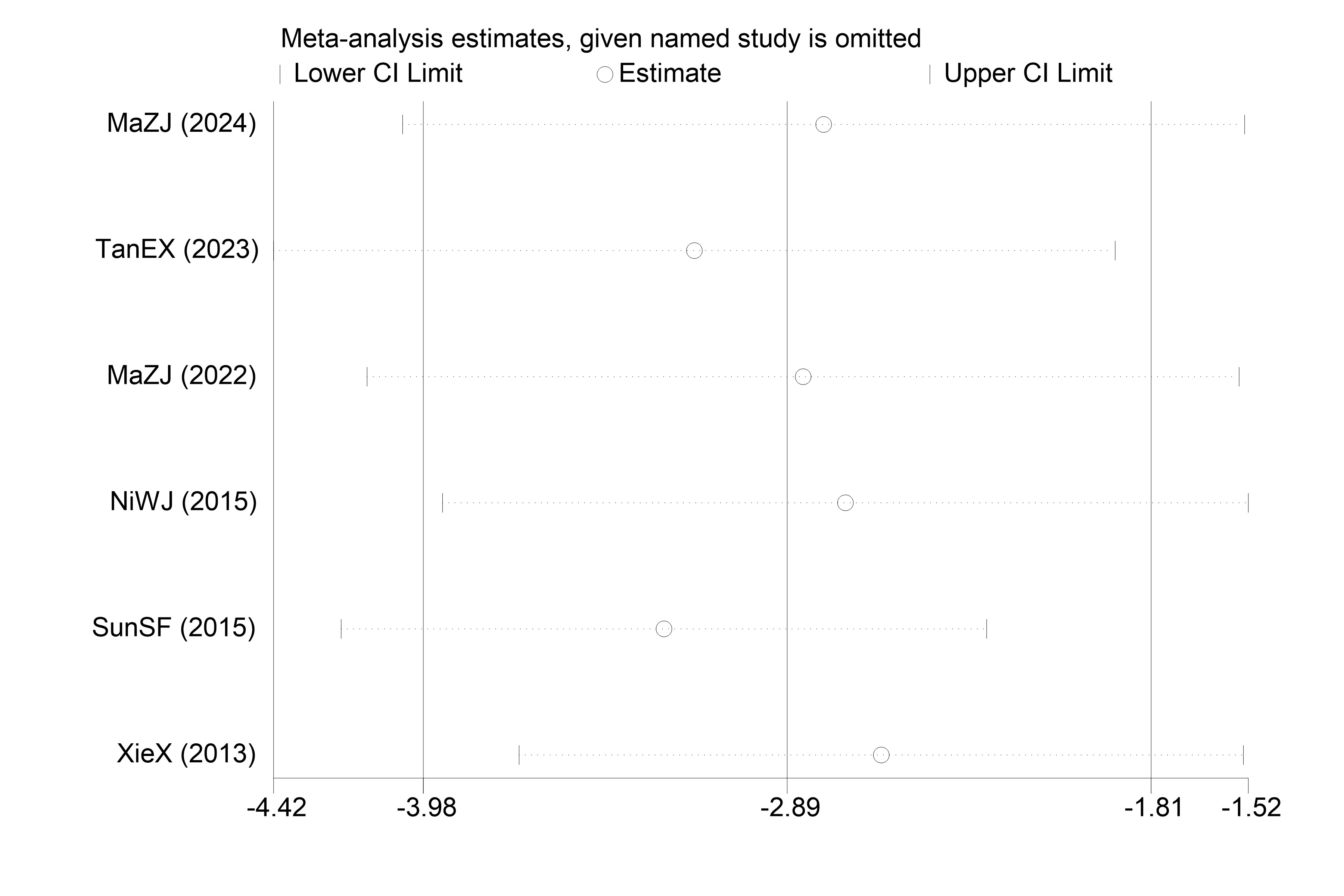** |
| Fibronectin**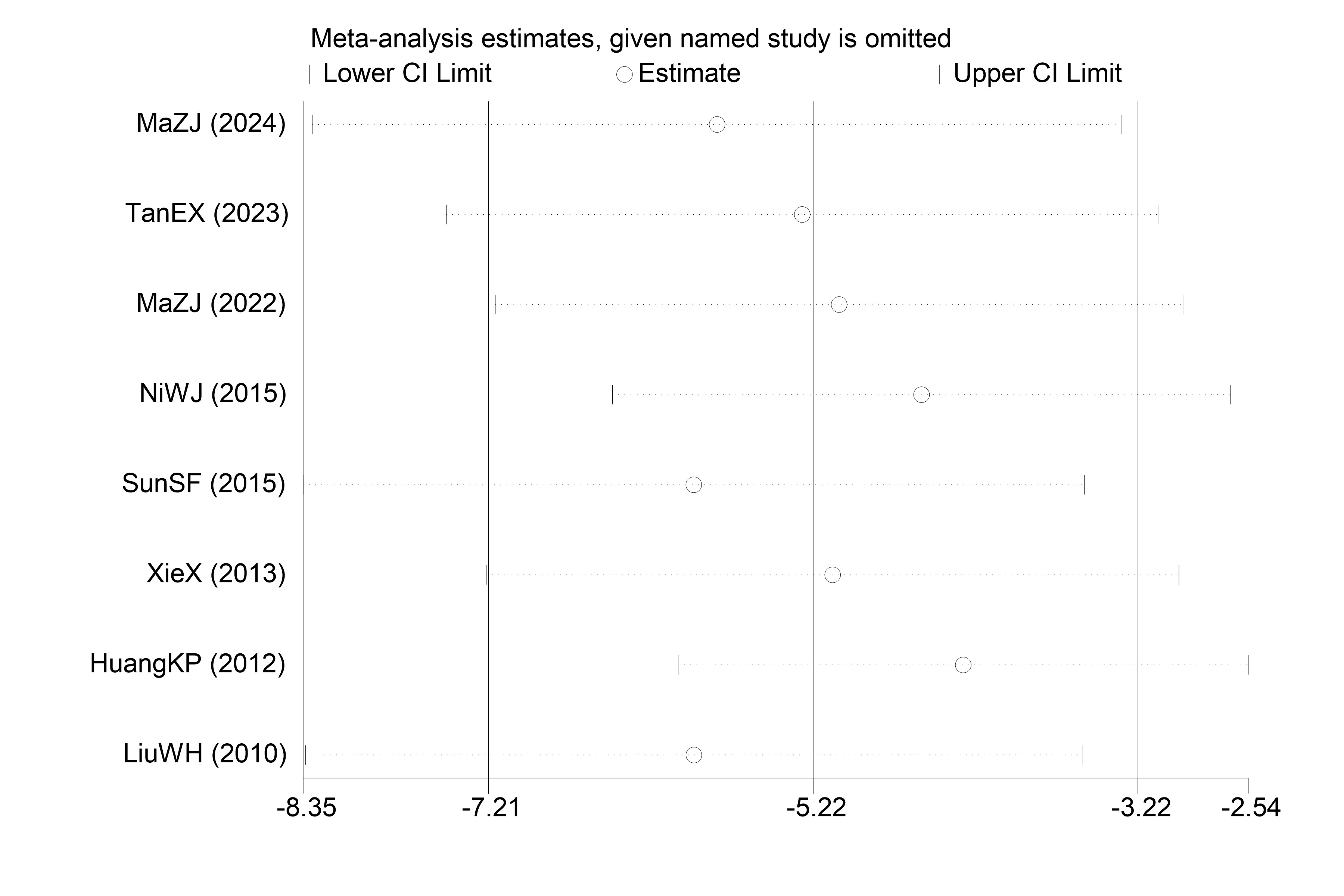** | E-cadherin**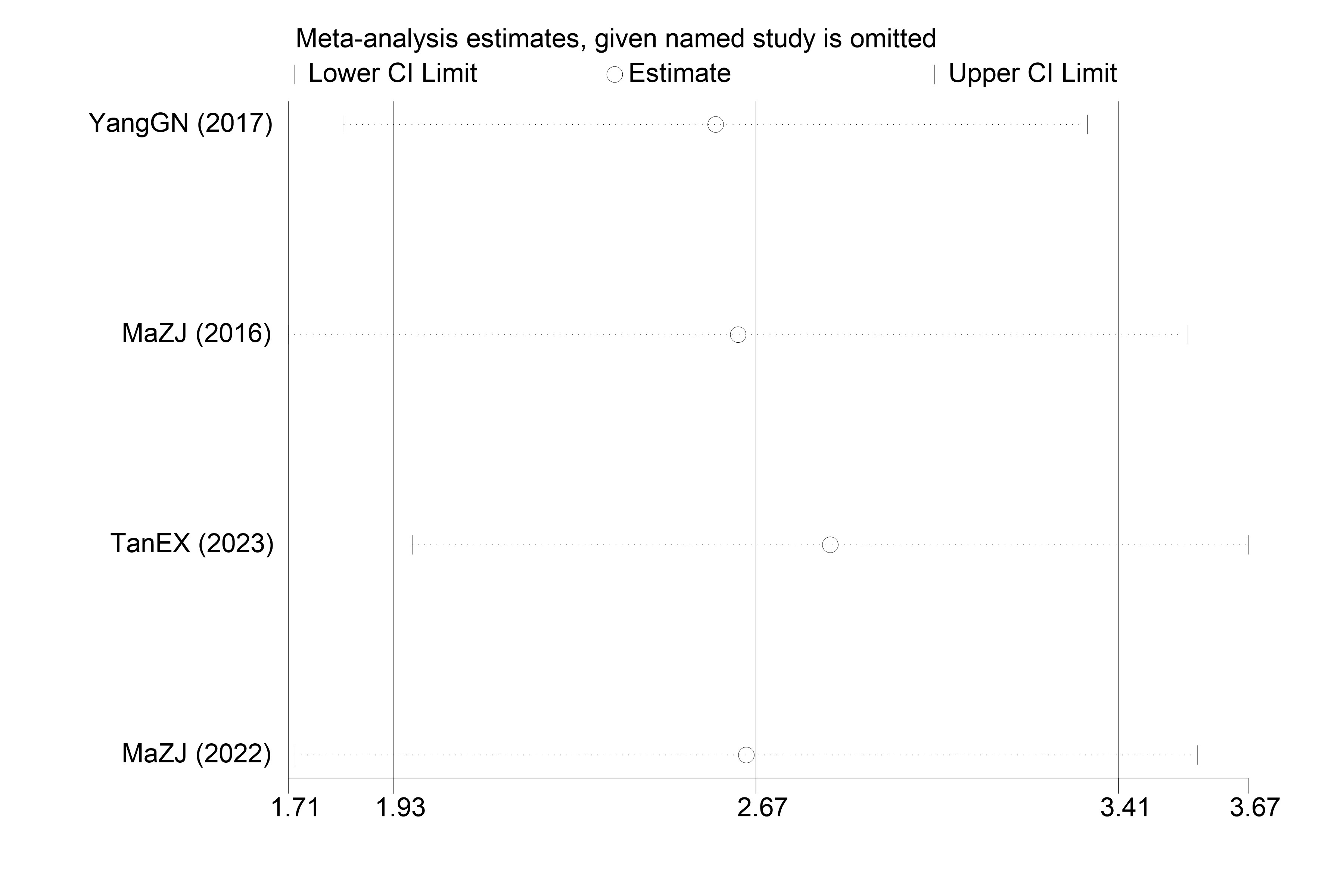** |
| Renal-fibrosis-area**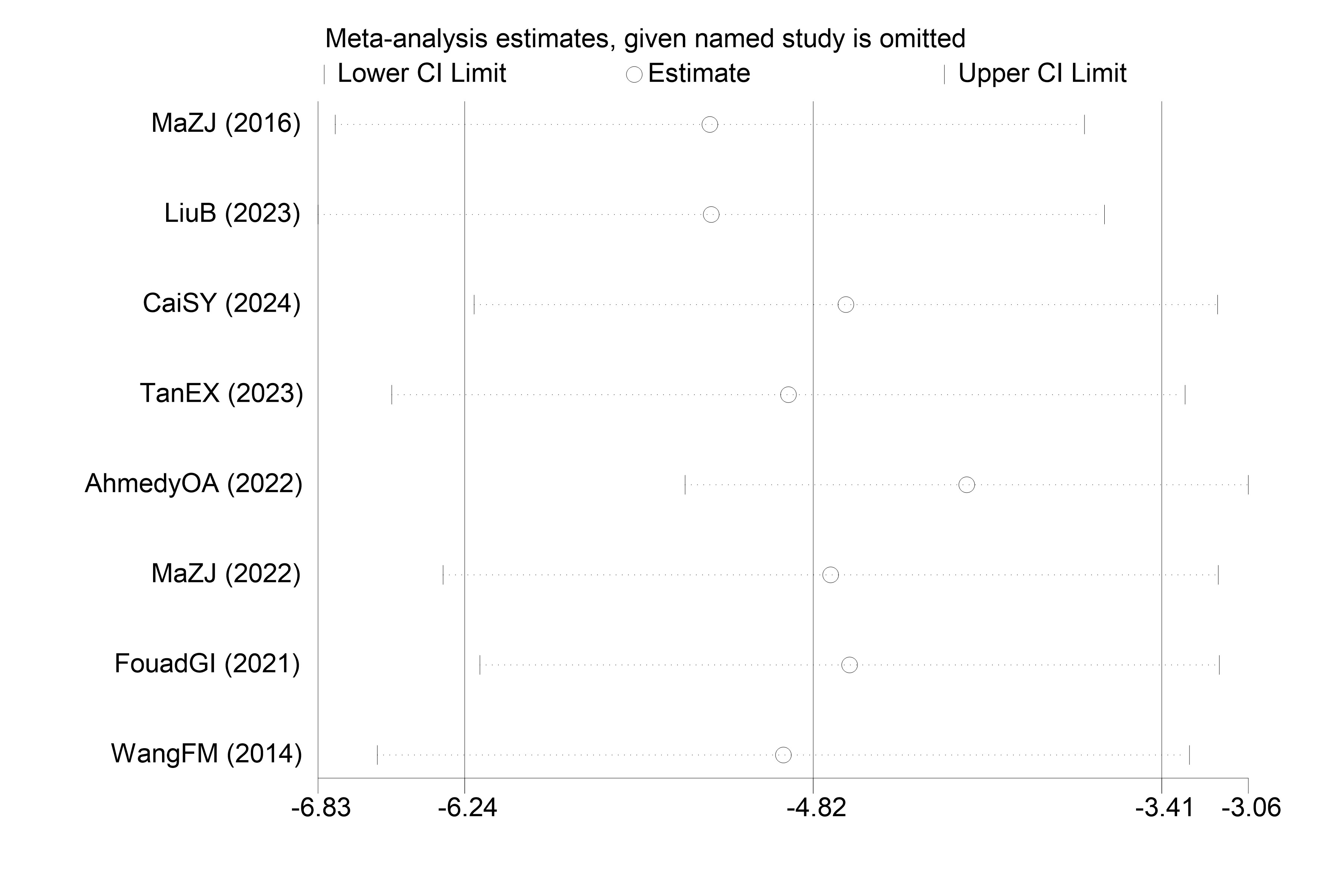** | KIM-1**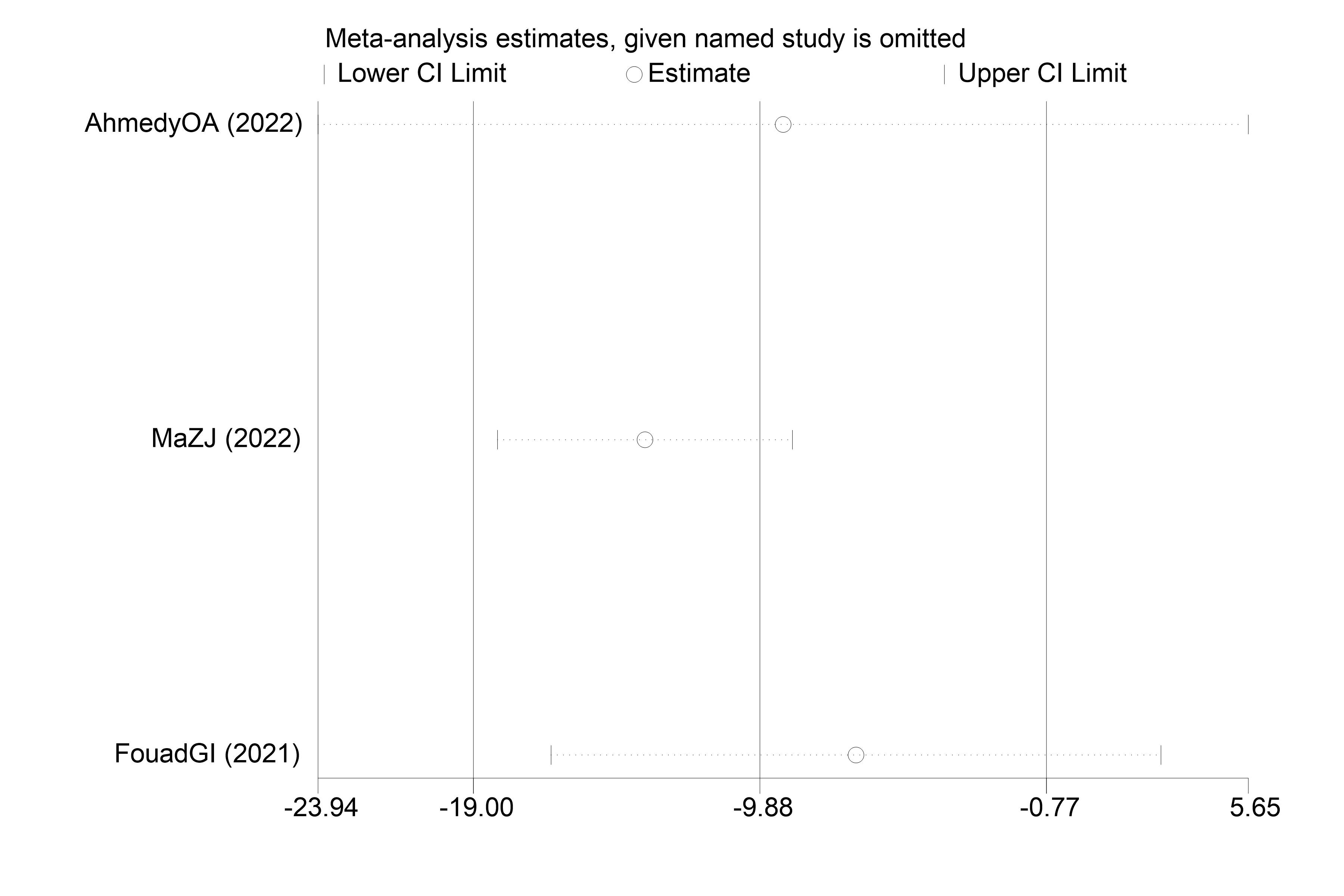** |
| Kidney-injury-score**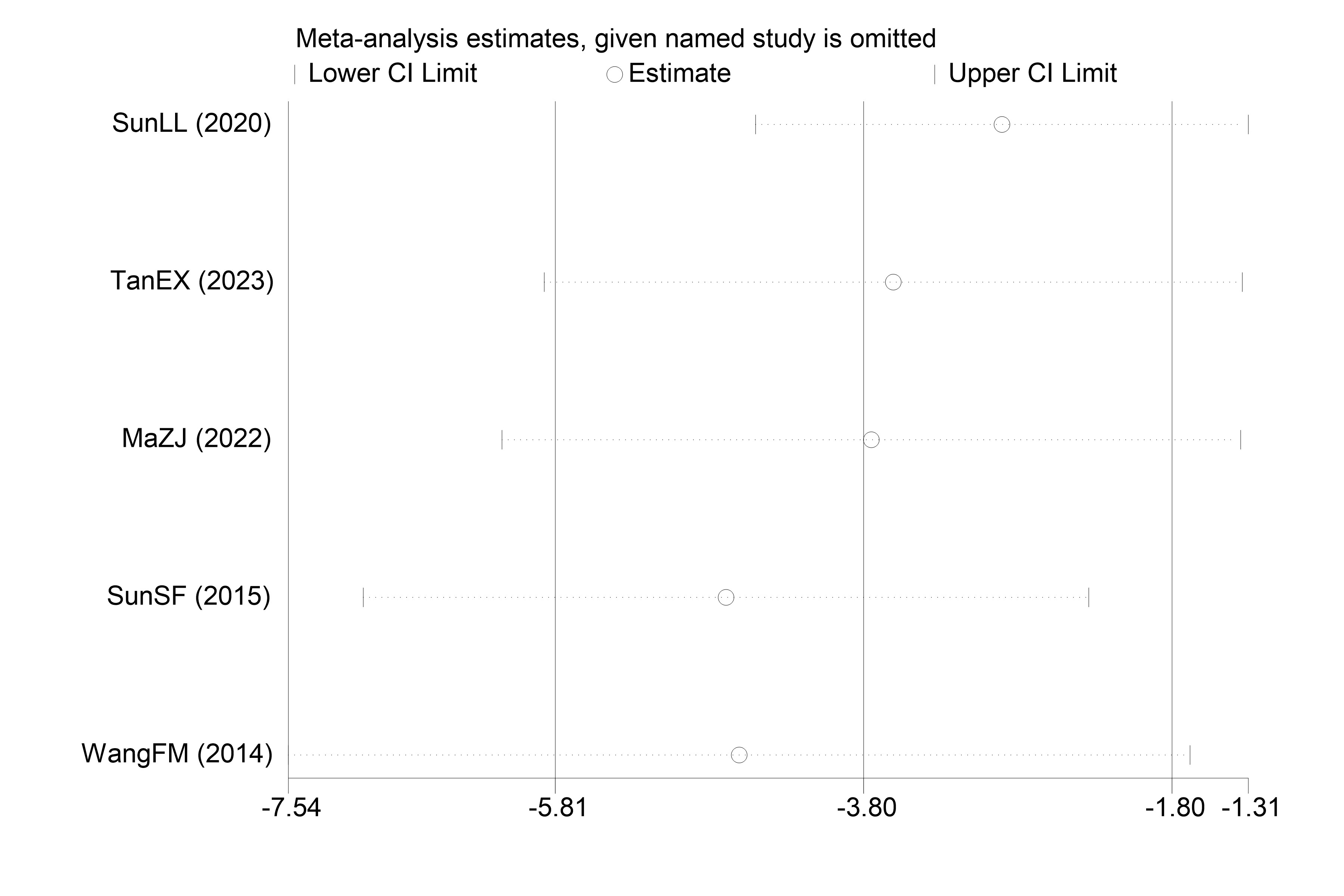** | MDA**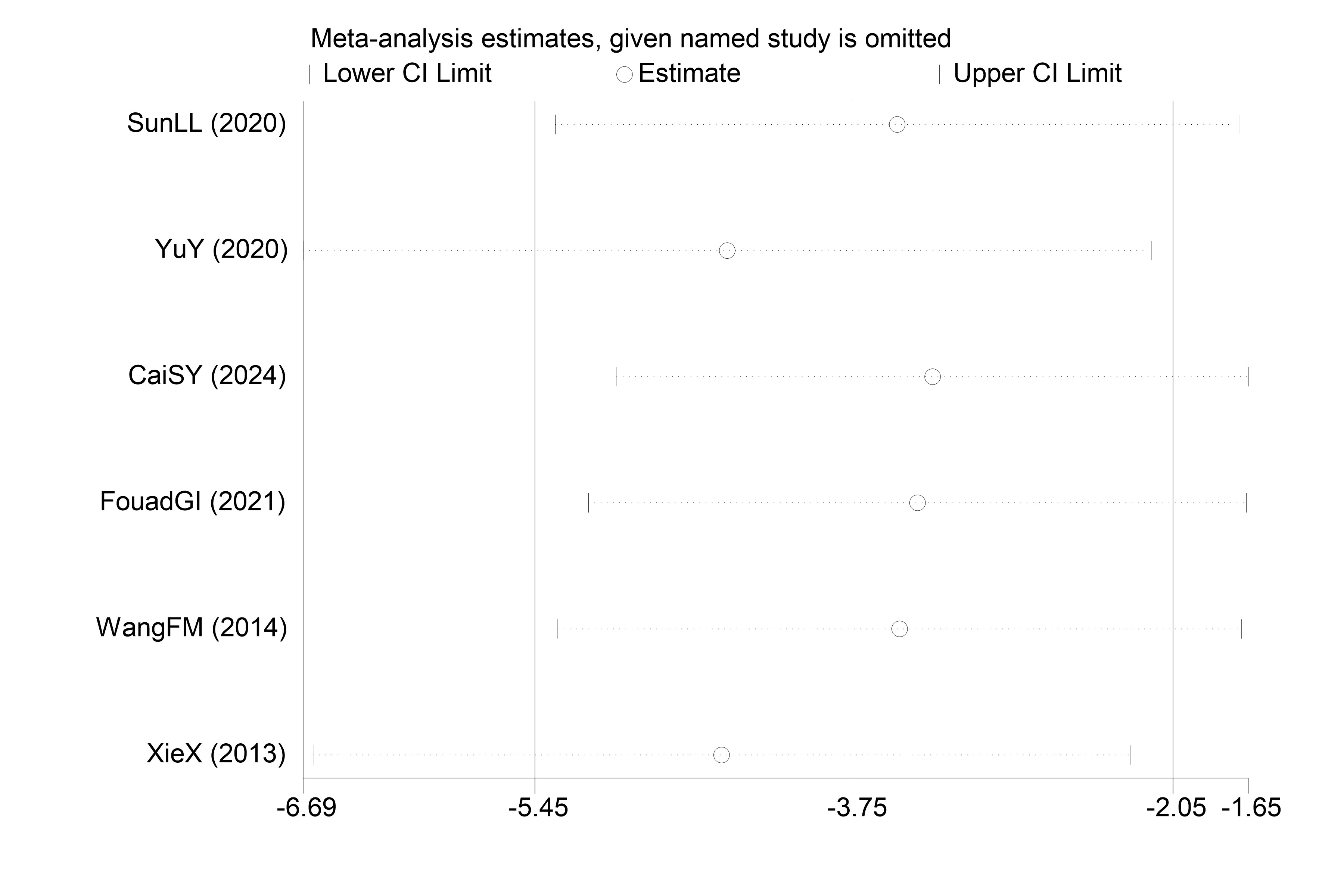** |
| SOD  **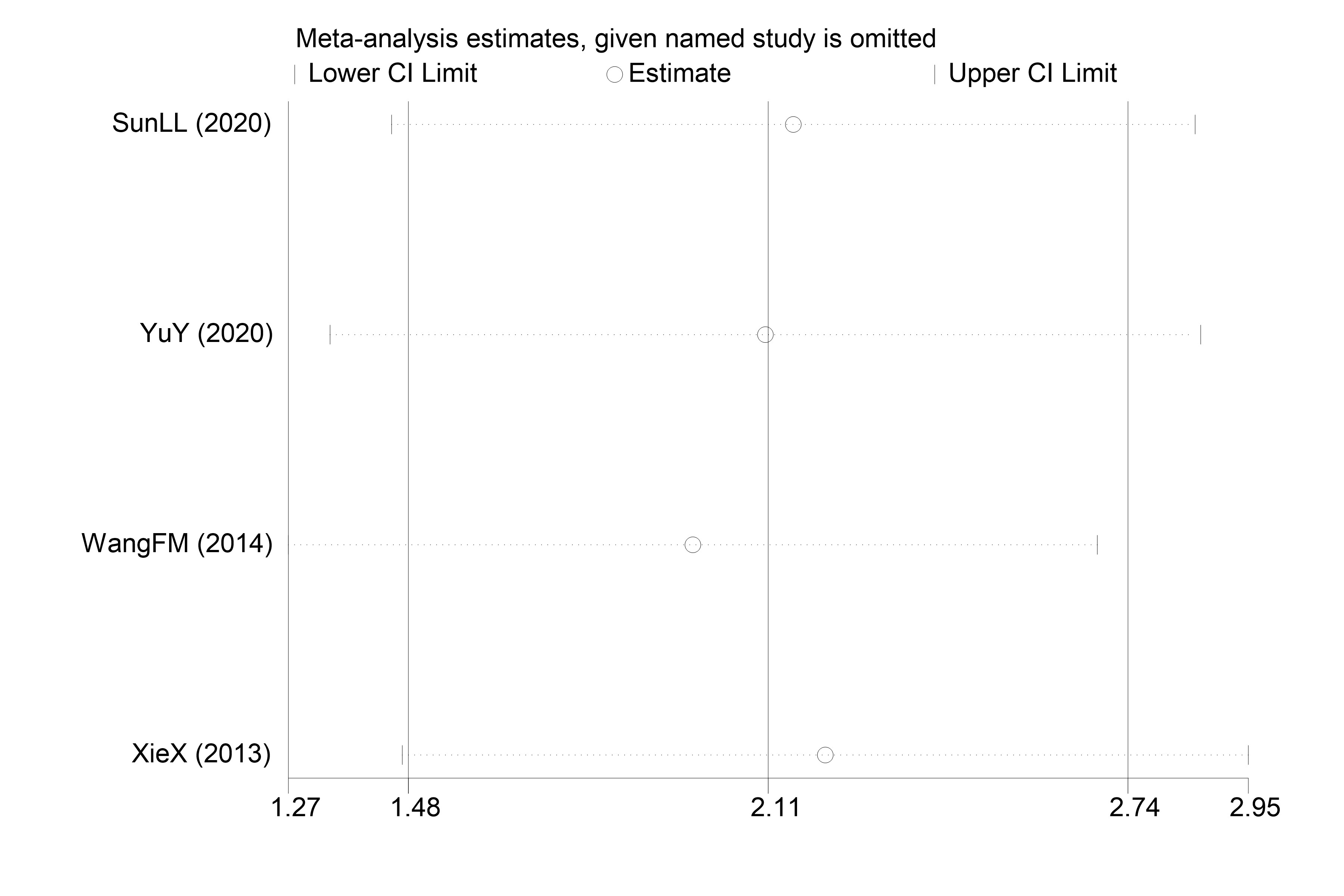** | GSH-Px  **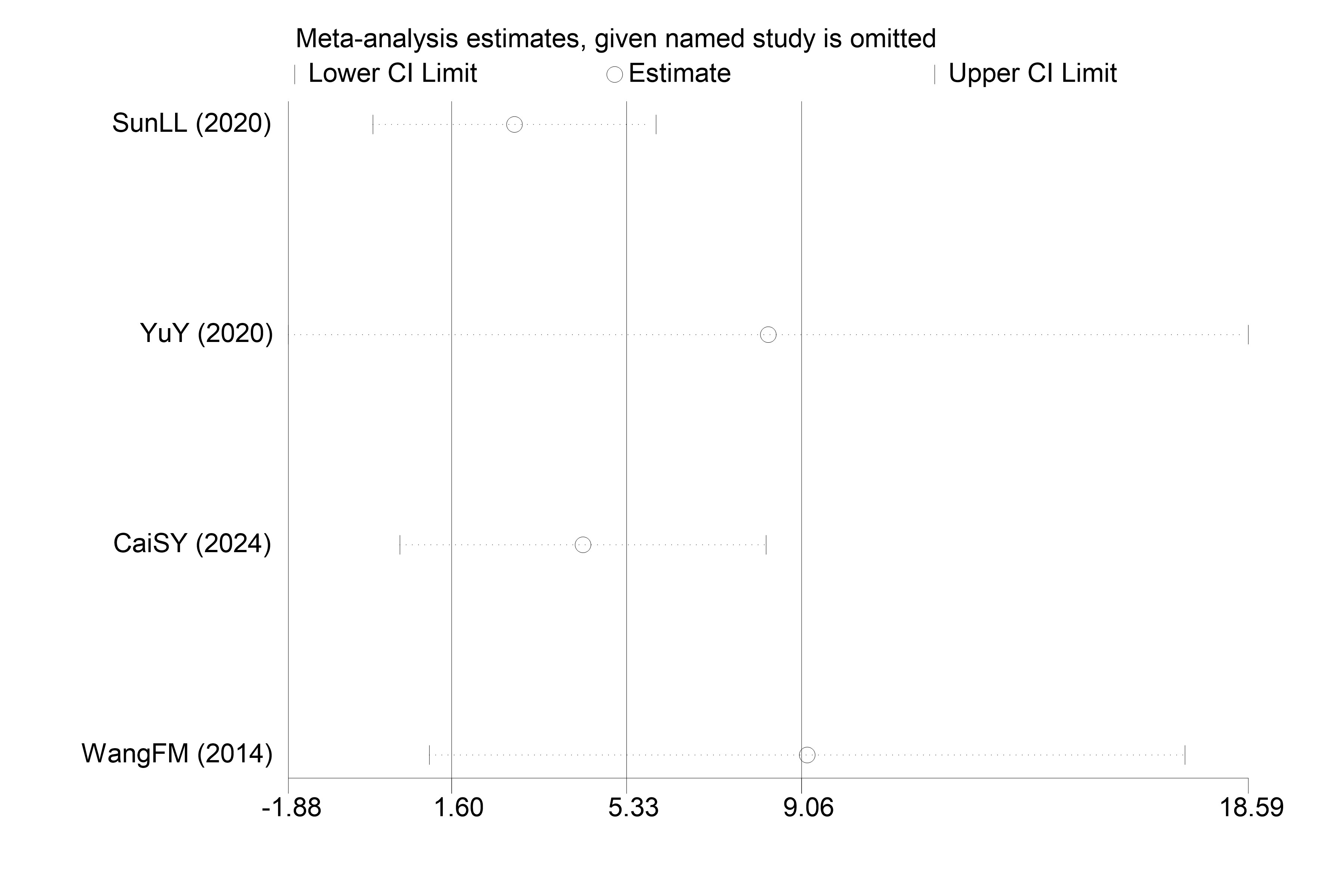** |
| IL-1β  **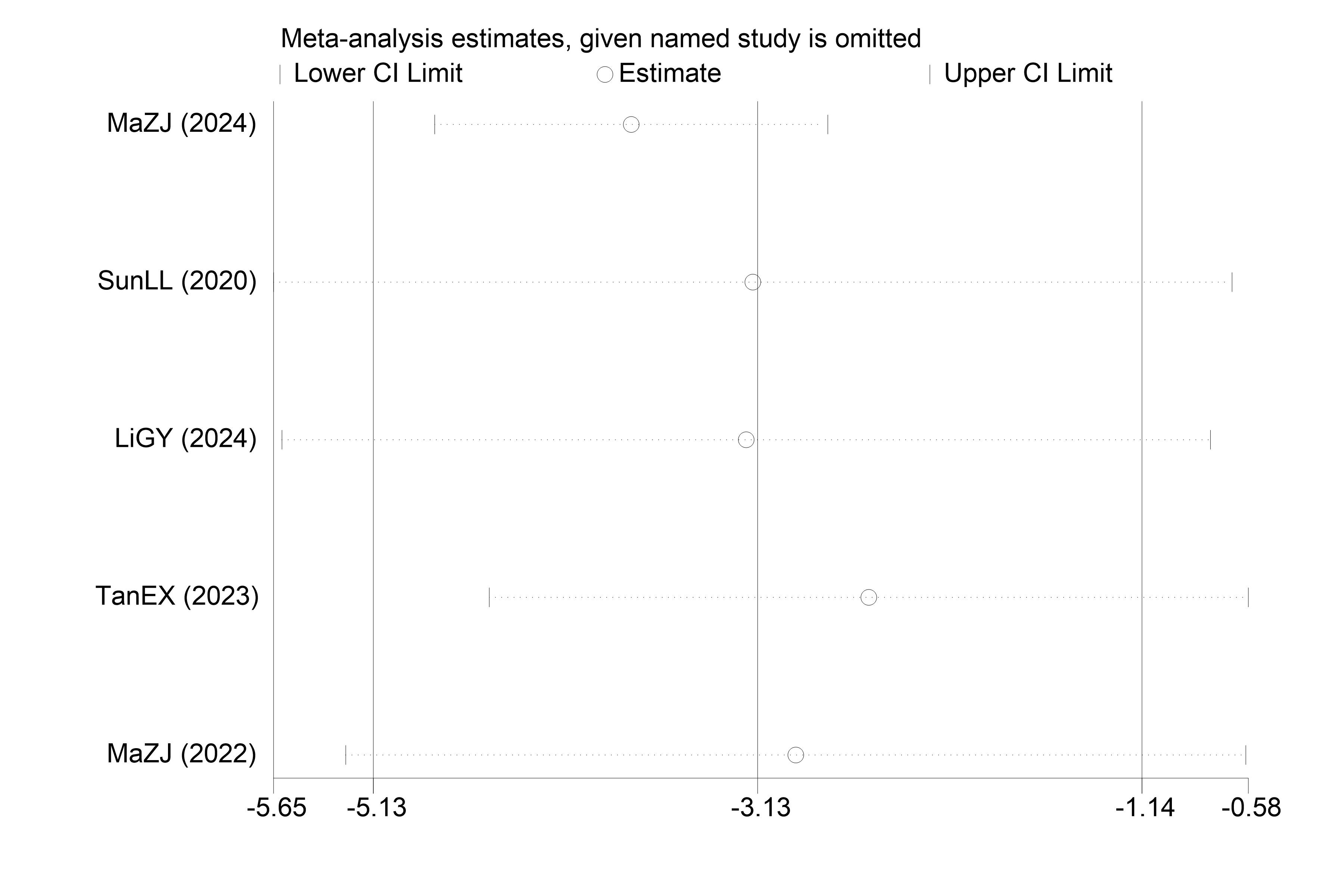** | NLRP3  **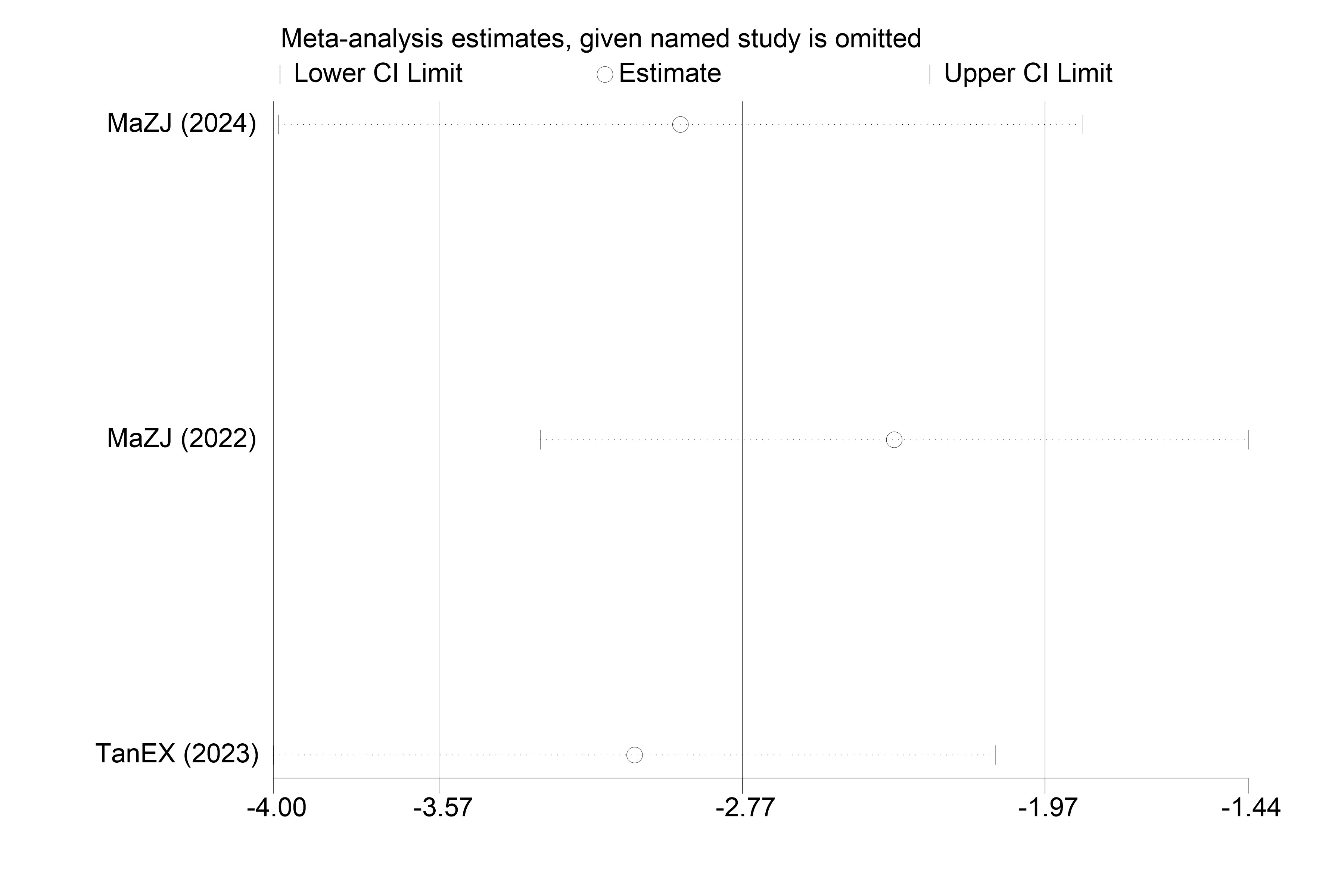** |

**Appendix Figure 2.** Funnel plots on Scr, BUN, TGF-β1, and 24h urine protein

**
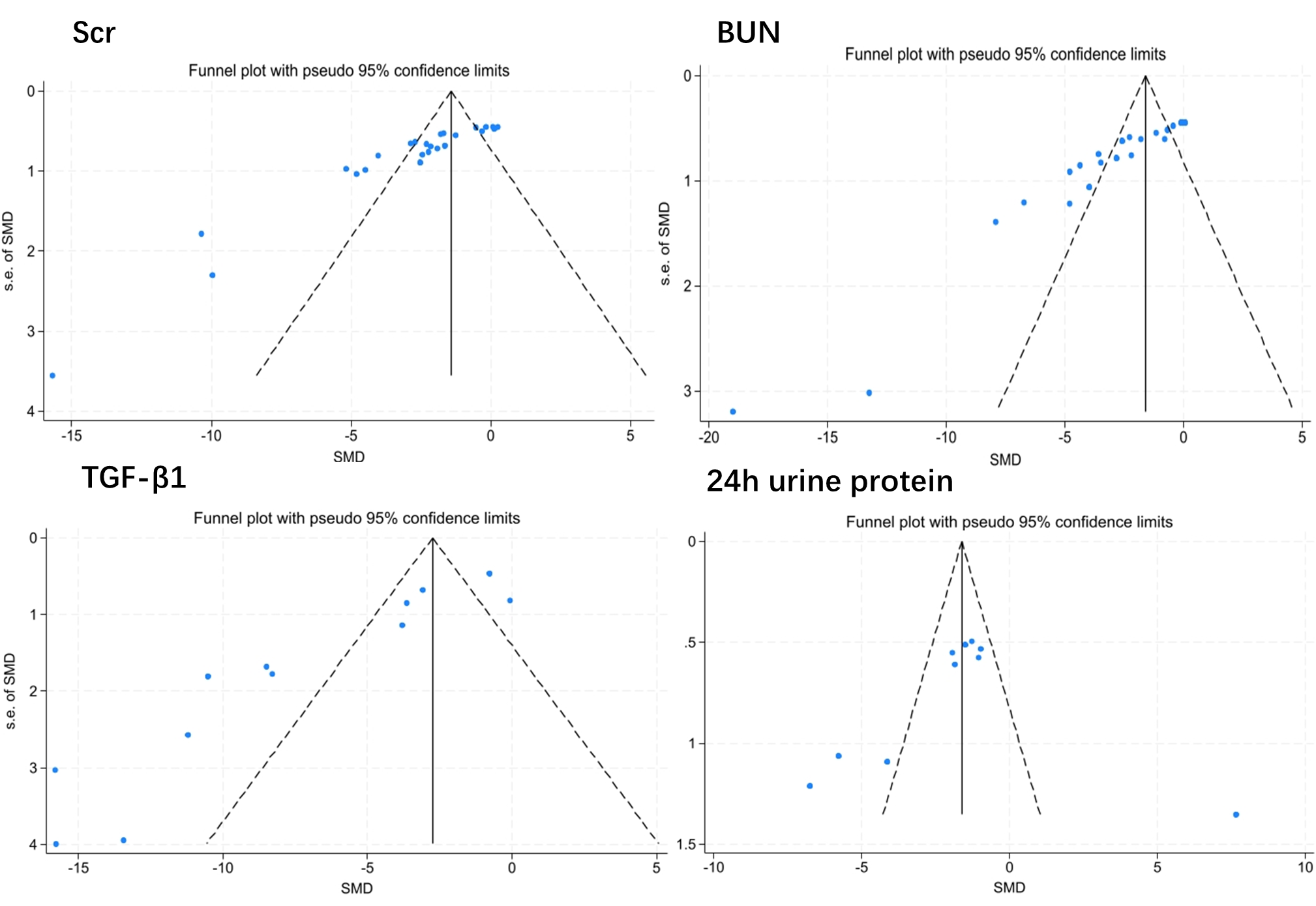
**

Figure 2. Funnel plots on Scr, BUN, TGF-β1, and 24h urine protein

# Supplementary Table

**Supplementary Table S1.** Literature search strategy for BBR in the treatment of Kidney Fibrosis

| Database | Number | Search terms |
| --- | --- | --- |
| **PubMed** | #1 | (Berberine[Title/Abstract]) OR (Umbellatine[Title/Abstract]) OR (berberine[Title/Abstract]) OR (berberine hydrochloride[Title/Abstract]) OR ("Berberine"[Mesh]) |
|  | #2 | (renal fibrosis[Title/Abstract]) OR (renal fibroblast[Title/Abstract]) OR (kidney fibrosis[Title/Abstract]) OR (kidney fibroblast[Title/Abstract]) OR (fibrosis[Title/Abstract]) OR (fibroblast[Title/Abstract]) OR (Chronic Renal Insufficiencies[Title/Abstract]) OR (Renal Insufficiency, Chronic[Title/Abstract]) OR (Renal Insufficiencies, Chronic[Title/Abstract]) OR (Chronic Renal Insufficiency[Title/Abstract]) OR (Chronic Kidney Insufficiency[Title/Abstract]) OR (Chronic Kidney Diseases[Title/Abstract]) OR (Chronic Renal Diseases[Title/Abstract]) OR ("Renal Insufficiency, Chronic"[Mesh]) |
|  | #3 | #1 AND #2 |
| **EMBASE** | #1 | 'berberine'/exp |
|  | #2 | 'berberine':ab,ti OR 'umbellatine':ab,ti OR 'berberine hydrochloride':ab,ti |
|  | #3 | #1 OR #2 |
|  | #4 | 'renal insufficiency, chronic'/exp |
|  | #5 | 'renal fibrosis':ab,ti OR 'renal fibroblast':ab,ti OR 'kidney fibrosis':ab,ti OR 'kidney fibroblast':ab,ti OR 'fibrosis':ab,ti OR 'fibroblast':ab,ti OR 'chronic renal insufficiencies':ab,ti OR 'renal insufficiencies, chronic':ab,ti OR 'chronic renal insufficiency':ab,ti OR 'chronic kidney insufficiency':ab,ti OR 'chronic kidney diseases':ab,ti OR 'chronic renal diseases':ab,ti OR 'renal insufficiency, chronic':ab,ti |
|  | #6 | #4 OR #5 |
|  | #7 | #3 AND #6 |
| **Web of Science** | #1 | Berberine (Topic) OR Umbellatine (Topic) OR berberine (Topic) OR berberine hydrochloride (Topic) |
|  | #2 | renal fibrosis (Topic) OR renal fibroblast (Topic) OR kidney fibroblast (Topic) OR kidney fibrosis (Topic) OR fibrosis (Topic) OR fibroblast (Topic) OR Chronic Renal Insufficiencies (Topic) OR Renal Insufficiency, Chronic (Topic) OR Renal Insufficiencies, Chronic (Topic) OR Chronic Renal Insufficiency (Topic) OR Chronic Kidney Insufficiency (Topic) OR Chronic Kidney Diseases (Topic) OR Chronic Renal Diseases (Topic) |
|  | #3 | #1 AND #2 |
| **SCIELO** | #1 | Berberine (Topic) OR Umbellatine (Topic) OR berberine (Topic) OR berberine hydrochloride (Topic) |
|  | #2 | renal fibrosis (Topic) OR renal fibroblast (Topic) OR kidney fibroblast (Topic) OR kidney fibrosis (Topic) OR fibrosis (Topic) OR fibroblast (Topic) OR Chronic Renal Insufficiencies (Topic) OR Renal Insufficiency, Chronic (Topic) OR Renal Insufficiencies, Chronic (Topic) OR Chronic Renal Insufficiency (Topic) OR Chronic Kidney Insufficiency (Topic) OR Chronic Kidney Diseases (Topic) OR Chronic Renal Diseases (Topic) |
|  | #3 | #1 AND #2 |
| **Scopus** |  | ( TITLE-ABS-KEY ( berberine ) OR TITLE-ABS-KEY ( umbellatine ) OR TITLE-ABS-KEY ( berberine ) OR TITLE-ABS-KEY ( "berberine hydrochloride" ) ) AND ( TITLE-ABS-KEY ( "renal fibrosis" ) OR TITLE-ABS-KEY ( "renal fibroblast" ) OR TITLE-ABS-KEY ( "kidney fibrosis" ) OR TITLE-ABS-KEY ( "kidney fibroblast" ) OR TITLE-ABS-KEY ( fibrosis ) OR TITLE-ABS-KEY ( fibroblast ) OR TITLE-ABS-KEY ( "Chronic Renal Insufficiencies" ) OR TITLE-ABS-KEY ( "Renal Insufficiency, Chronic" ) OR TITLE-ABS-KEY ( "Renal Insufficiencies, Chronic" ) OR TITLE-ABS-KEY ( "Chronic Renal Insufficiency" ) OR TITLE-ABS-KEY ( "Chronic Kidney Insufficiency" ) OR TITLE-ABS-KEY ( "Chronic Kidney Diseases" ) OR TITLE-ABS-KEY ( "Chronic Renal Diseases" ) ) |
| **Cochrane Library** | #1 | MeSH descriptor: [Berberine] explode all trees |
|  | #2 | (Berberine):ti,ab,kw OR (Umbellatine):ti,ab,kw OR (berberine):ti,ab,kw OR (berberine hydrochloride):ti,ab,kw |
|  | #3 | #1 OR #2 |
|  | #4 | MeSH descriptor: [Renal Insufficiency, Chronic] explode all trees |
|  | #5 | (renal fibrosis):ti,ab,kw OR (renal fibroblast):ti,ab,kw OR (kidney fibrosis):ti,ab,kw OR (kidney fibroblast):ti,ab,kw OR (fibrosis):ti,ab,kw |
|  | #6 | (fibroblast):ti,ab,kw OR (Chronic Renal Insufficiencies):ti,ab,kw OR (Renal Insufficiency, Chronic):ti,ab,kw OR (Renal Insufficiencies, Chronic):ti,ab,kw OR (Chronic Renal Insufficiency):ti,ab,kw |
|  | #7 | (Chronic Kidney Insufficiency):ti,ab,kw OR (Chronic Kidney Diseases):ti,ab,kw OR (Chronic Renal Diseases):ti,ab,kw |
|  | #8 | #4 OR #5 OR #6 OR #7 |
|  | #9 | #3 AND #8 |
| **CNKI** |  | （主题：纤维化）OR（主题：肾纤维化）OR（主题：慢性肾功能不全）OR（主题：慢性肾脏功能不全）OR（主题：慢性肾病）AND（主题：小檗碱）OR（主题：盐酸小檗碱）OR（主题：黄连素） |
| **WangFang database** |  | (小檗碱 OR 盐酸小檗碱 OR 黄连素) and 主题:(纤维化 OR 肾纤维化 OR 慢性肾功能不全 OR 慢性肾脏功能不全 OR 慢性肾病) |
| **VIP** |  | (U=小檗碱 OR U=盐酸小檗碱 OR U=黄连素) AND (U=纤维化 OR U=肾纤维化 OR U=慢性肾功能不全 OR U=慢性肾脏功能不全 OR U=慢性肾病) |
| **CBM** | #1 | "小檗碱"[不加权:扩展] |
|  | #2 | "小檗碱"[常用字段:智能] OR "盐酸小檗碱"[常用字段:智能] OR "黄连素"[常用字段:智能] |
|  | #3 | (#2) OR (#1) |
|  | #4 | "纤维化"[不加权:扩展] |
|  | #5 | "肾纤维化"[常用字段:智能] OR "纤维化"[常用字段:智能] |
|  | #6 | "肾功能不全, 慢性"[不加权:扩展] |
|  | #7 | "慢性肾功能不全"[常用字段:智能] OR "慢性肾脏功能不全"[常用字段:智能] OR "慢性肾病"[常用字段:智能] |
|  | #8 | (#9) OR (#8) OR (#5) OR (#4) |
|  | #9 | (#10) AND (#3) |

**Supplementary Table S2.** The methodological quality of included studies

| Study(year) | **1** | **2** | **3** | **4** | **5** | **6** | **7** | **8** | **9** | **10** |
| --- | --- | --- | --- | --- | --- | --- | --- | --- | --- | --- |
| LiuWH et al(2010) | **?** | **+** | **?** | **?** | **?** | **?** | **?** | **+** | **+** | **+** |
| HuangKP et al(2012) | **?** | **+** | **?** | **?** | **?** | **?** | **?** | **+** | **+** | **+** |
| LiuS et al(2012) | **?** | **+** | **?** | **+** | **?** | **?** | **?** | **-** | **+** | **+** |
| XieX et al(2013) | **?** | **+** | **?** | **+** | **?** | **?** | **?** | **+** | **+** | **+** |
| WangFM et al(2014) | **?** | **+** | **?** | **?** | **?** | **?** | **?** | **+** | **+** | **+** |
| MiaoGG et al(2014) | **?** | **+** | **?** | **?** | **?** | **?** | **?** | **+** | **+** | **+** |
| NiWJ et al(2015) | **?** | **+** | **?** | **+** | **?** | **?** | **?** | **+** | **+** | **+** |
| SunSF et al(2015) | **?** | **+** | **?** | **+** | **?** | **?** | **?** | **+** | **+** | **+** |
| ZhangXL et al(2016) | **?** | **+** | **?** | **?** | **?** | **?** | **?** | **+** | **+** | **+** |
| MaZJ et al(2016) | **+** | **+** | **?** | **+** | **?** | **?** | **?** | **+** | **+** | **+** |
| QiuYY et al(2017) | **?** | **+** | **?** | **+** | **?** | **?** | **?** | **-** | **+** | **+** |
| YangGN et al(2017) | **?** | **+** | **?** | **?** | **?** | **?** | **?** | **-** | **+** | **+** |
| LiZ et al(2017) | **?** | **+** | **?** | **+** | **?** | **?** | **?** | **+** | **+** | **+** |
| SunLL et al(2020) | **+** | **+** | **?** | **?** | **?** | **?** | **?** | **+** | **+** | **+** |
| YuY et al(2020) | **?** | **+** | **?** | **?** | **?** | **?** | **?** | **+** | **+** | **+** |
| XiaoYP et al(2021) | **?** | **+** | **?** | **+** | **?** | **?** | **?** | **+** | **+** | **+** |
| FouadGI et al(2021) | **?** | **+** | **?** | **+** | **?** | **?** | **?** | **+** | **+** | **+** |
| MaZJ et al(2022) | **+** | **+** | **?** | **+** | **?** | **?** | **?** | **+** | **+** | **+** |
| AhmedyOA et al(2022) | **?** | **+** | **?** | **+** | **?** | **?** | **?** | **+** | **+** | **+** |
| TanEX et al(2023) | **?** | **+** | **+** | **?** | **?** | **?** | **?** | **+** | **+** | **+** |
| Al-jebouriDB et al(2023) | **?** | **+** | **?** | **?** | **?** | **?** | **?** | **+** | **+** | **+** |
| WangYJ et al(2023) | **?** | **+** | **?** | **?** | **?** | **?** | **?** | **-** | **+** | **+** |
| LiuB et al(2023) | **?** | **+** | **?** | **?** | **?** | **?** | **?** | **+** | **+** | **+** |
| CaiSY et al(2024) | **?** | **+** | **?** | **?** | **?** | **?** | **?** | **-** | **+** | **+** |
| MaZJ et al(2024) | **+** | **+** | **?** | **+** | **?** | **?** | **?** | **+** | **+** | **+** |
| LiGY et al(2024) | **?** | **+** | **?** | **?** | **?** | **?** | **?** | **-** | **+** | **+** |

1) sequence generation; 2) baseline characteristics; 3) allocation concealment; 4) random housing; 5) blinding (caregivers/investigators); 6) randomization for outcome assessment; 7) blinding (outcome assessor); 8) incomplete outcome data; 9) selective outcome reporting; 10) other biases. +, low risk of bias; −, high risk of bias; ?, unclear risk of bias

**Supplementary Table S3.** The subgroup analysis of BBR on Scr, BUN, TGF-β1 and α-SMA

| Parameter | Subgroup | | No.studies | SMD[95% CI] | I^2^ | Q;df;P |
| --- | --- | --- | --- | --- | --- | --- |
| Scr | Publication year | Before 2017 | 13 | -2.16(-3.10,-1.22) | 86.5% | 0.28;1;0.595 |
|  |  | After 2017 | 12 | -2.64(-3.77,-1.52) | 86.4% |  |
|  | Animals pecies | Rats | 17 | -2.17(-3.02,-1.31) | 87.2% | 5.66;1;0.017 |
|  |  | Mice |  | -2.79(-4.06,-1.52) | 82.5% |  |
|  | Drug  dosage | ＜200mg/kg | 10 | -0.96(-1.74,-0.18) | 78.4% | 52.39;1;0.000 |
|  |  | ≥200mg/kg | 15 | -3.20(-4.11,-2.29) | 81.7% |  |
|  | Treatment  duration | ＞8W | 10 | -1.60(-2.52,-0.67) | 84.8% | 9.80;1;0.002 |
|  |  | ≤8W | 15 | -3.02(-4.07,-1.98) | 86.2% |  |
|  | Induction  methods | DN model | 17 | -2.28(-3.15,-1.42) | 87.6% | 35.14;6;0.000 |
|  |  | I/R Model | 1 | -15.69(-22.66,-8.72) | / |  |
|  |  | Unilateral Renal Artery Stenosis Model | 1 | -2.52(-4.27,-0.77) | / |  |
|  |  | UUO Model | 3 | -1.36(-2.63,-0.08) | 66.3% |  |
|  |  | Adenine-Induced Chronic Renal Failure Model | 1 | -1.79(-2.85,-0.74) | / |  |
|  |  | Cisplatin induced renal fibrosis model | 1 | -5.17(-7.08,-3.27) | / |  |
|  |  | Doxorubicin induced renal fibrosis model | 1 | -1.91(-3.32,-0.50) | / |  |
| BUN | Publication year | Before 2017 | 12 | -2.89(-4.08,-1.71) | 89.4% | 0.00;1;0.949 |
|  |  | After 2017 | 10 | -3.03(-4.45,-1.61) | 89.0% |  |
|  | Animals pecies | Rats | 15 | -2.37(-3.35,-1.39) | 89.0% | 27.95;1;0.000 |
|  |  | Mice | 7 | -4.12(-5.70,-2.54) | 80.4% |  |
|  | Drug  dosage | ＜200mg/kg | 9 | -2.49(-3.89,-1.10) | 90.1% | 24.58;1;0.000 |
|  |  | ≥200mg/kg | 13 | -3.17(-4.23,-2.11) | 85.1% |  |
|  | Treatment  duration | ＞8W | 9 | -2.33(-3.55,-1.10) | 89.0% | 7.00;1;0.008 |
|  |  | ≤8W | 13 | -3.47(-4.76,-2.18) | 88.6% |  |
|  | Induction  methods | DN model | 15 | -2.48(-3.48,-1.48) | 89.2% | 47.22;5;0.000 |
|  |  | I/R Model | 1 | -13.25(-19.17,-7.33) | / |  |
|  |  | UUO Model | 3 | -2.11(-3.90,-0.32) | 77.2% |  |
|  |  | Adenine-Induced Chronic Renal Failure Model | 1 | -4.37(-6.04,-2.69) | / |  |
|  |  | Cisplatin induced renal fibrosis model | 1 | -4.79(-6.59,-3.00) | / |  |
|  |  | Doxorubicin induced renal fibrosis model | 1 | -4.80(-7.19,-2.41) | / |  |
| TGF-β1 | Publication year | Before 2017 | 8 | -7.53(-10.08,-4.97) | 85.5% | 42.55;1;0.000 |
|  |  | After 2017 | 4 | -4.32(-7.85,-0.79) | 89.0% |  |
|  | Animals pecies | Rats | 9 | -7.72(-10.61,-4.84) | 91.9% | 2;1;0.157 |
|  |  | Mice | 3 | -3.88(-8.05,0.29) | 88.7% |  |
|  | Drug  dosage | ＜200mg/kg | 3 | -4.42(-9.19,0.36) | 90.4% | 1.95;1;0.162 |
|  |  | ≥200mg/kg | 9 | -7.64(-10.41,-4.87) | 91.6% |  |
|  | Treatment  duration | ＞8W | 3 | -5.85(-9.49,-2.22) | 88.1% | 4.54;1;0.033 |
|  |  | ≤8W | 9 | -7.08(-10.12,-4.04) | 91.7% |  |
|  | Induction  methods | DN model | 7 | -6.96(-10.07,-3.84) | 91.0% | 50.51;4;0.000 |
|  |  | Unilateral Renal Artery Stenosis Model | 1 | -3.80(-6.03,-1.56) | / |  |
|  |  | UUO Model | 2 | -9.68(-13.82,-5.55) | 24.8% |  |
|  |  | Adenine-Induced Chronic Renal Failure Model | 1 | -0.78(-1.69,0.14) | / |  |
|  |  | Doxorubicin induced renal fibrosis model | 1 | -11.21(-16.25,-6.17) | / |  |
| α-SMA | Publication year | Before 2017 | 5 | -5.00(-7.87,-2.12) | 90.2% | 2.44;1;0.118 |
|  |  | After 2017 | 3 | -3.20(-4.37,-2.03) | 42.8% |  |
|  | Animals pecies | Rats | 5 | -4.36(-6.53,-2.19) | 89.1% | 0.80;1;0.372 |
|  |  | Mice | 3 | -3.29(-6.42,-0.17) | 78.6% |  |
|  | Drug  dosage | ＜200mg/kg | 4 | -7.13(-11.05,-3.21) | 85.0% | 16.11;1;0.000 |
|  |  | ≥200mg/kg | 4 | -2.32(-3.51,-1.12) | 71.9% |  |
|  | Treatment  duration | ＞8W | 4 | -2.58(-4.44,-0.72) | 80.4% | 7.40;1;0.007 |
|  |  | ≤8W | 4 | -5.28(-8.17,-2.39) | 87.5% |  |
|  | Induction  methods | DN model | 5 | -4.58(-7.21,-1.94) | 89.8% | 6.46;2;0.040 |
|  |  | UUO Model | 2 | -4.27(-5.83,-2.72) | 15.3% |  |
|  |  | Adenine-Induced Chronic Renal Failure Model | 1 | -2.33(-3.49,-1.17) | / |  |

**Supplementary Table S4.** Abbreviations

| **Abbreviations**: | |
| --- | --- |
| ACSL4: Acyl-CoA synthetase long-chain family member 4 | AGEs: advanced glycation end products |
| BBR: Berberine | BUN: Blood urea nitrogen |
| CAT: Catalase | CKD: Chronic kidney disease |
| DN: Diabetic nephropathy | ECM: Extracellular matrix |
| EMT: Epithelial-mesenchymal transition | FN: Fibronectin |
| GPX4: Glutathione peroxidase 4 | GSH-Px: Glutathione peroxidase |
| HO-1: heme oxygenase-1 | IL-1β: Interleukin-1β |
| I/R: Ischemia-reperfusion | KIM-1: Kidney injury molecule-1 |
| KWI: Kidney weight index | MCP-1: Monocyte Chemotactic Protein 1 |
| MDA: Malondialdehyde | MMPs: matrix metalloproteinases |
| MRA: Mineralocorticoid receptor antagonists | NF-κB: nuclear factor kappa-B |
| NLRP3: NOD-like receptor protein 3 | NQO1: NAD(P)H quinone oxidoreductase 1 |
| PAS: Periodic Acid-Schiff | RAGE: receptor for advanced glycation end-products |
| RAS: Renin-angiotensin system | ROS: Reactive oxygen species |
| Scr: Serum creatinine | SOD: Superoxide dismutase |
| SphK1- S1P: sphingosine kinase 1 -sphingosine-1-phosphate | STZ: Streptozotocin |
| TGF-β1: Transforming growth factor-β1 | TIMPs: tissue inhibitors of metalloproteinases |
| TNF-α: Tumor necrosis factor-α | UUO: Unilateral ureteral obstruction |
| α-SMA: α-smooth muscle actin |  |
